# Nature of intermolecular interaction in squaraine dimers

**DOI:** 10.1038/s41598-020-76631-z

**Published:** 2020-11-12

**Authors:** Anna Kaczmarek-Kędziera, Piotr S. Żuchowski, Dariusz Kędziera

**Affiliations:** 1 Faculty of Chemistry, Nicolaus Copernicus University in Torun, Gagarina 7, 87-100 Toruń, Poland; 2 Institute of Physics, Nicolaus Copernicus University in Torun, Grudziadzka 5, 87-100 Toruń, Poland

**Keywords:** Physical chemistry, Theoretical chemistry

## Abstract

Squaraine dyes are known for their particular optical properties. They exhibit intense photochemically stable fluorescence in usually (near) infra red region that can be quenched by intermolecular interactions. Moreover, even the centrosymmetric dyes feature non-zero second harmonic generation upon aggregation. Therefore, the detailed knowledge of the squaraine dye interaction nature both in homogenic aggregates and with other species present in the environment can be of importance for the design of new materials of desired properties. In the present study, interaction in squaraine dimers is investigated with quantum chemistry tools. Four structures: two stacked and two hydrogen-bonded are analyzed in terms of supermolecular approach and symmetry-adapted perturbation theory. MP2C/aug-cc-pVTZ supermolecular calculations confirm the particular stability of the stacked dimers and the favoured dispersion attraction for the long-displaced system.

## Introduction

**Figure 1:**
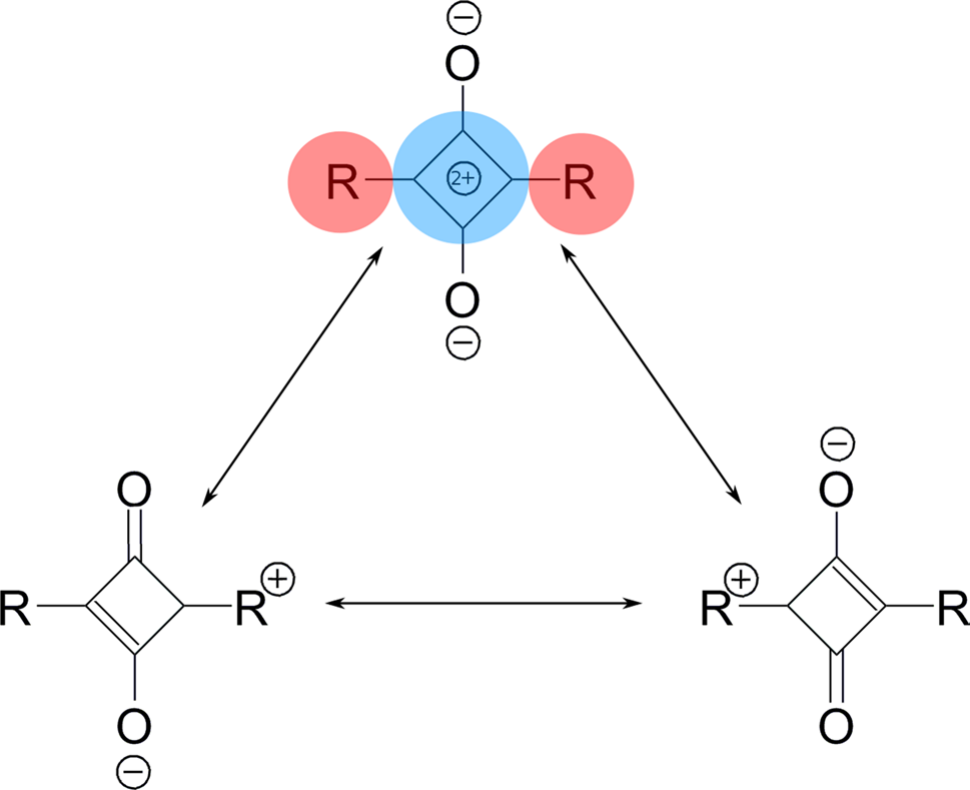
Resonance structures of symmetric squaraine molecule with red areas depicting the electron-rich moieties and blue areas—the electron-defficient group (color on-line).

Squaraine dyes belongs to the class of quadrupolar molecules of high interests in materials chemistry, nonlinear optics or photonics. Their peculiar photooptical properties arise from their unique structure: an electron-defficient four-membered squaric acid ring (denoted further by A as acceptor) is placed in between two electron-rich donating groups (denoted by D—see Scheme Fig. 1). This D-A-D structure results in a specific strong and sharp absorption in visible or near-IR region and interesting nonlinear properties. The properties of isolated molecules are significantly affected by self-aggregation of the dyes. The aggregation phenomena may occur in solution and in solid state and cause strong dipole coupling notably modyfiyng dye photophysical behaviour. The two limiting types of aggregates are described in literature: J-aggregates and H-aggregates. J-aggregates possess the head-to-tail transition dipole arrangement and exhibit the sharp and intense red-shifted absorption band. They are also the good fluorescent agents. On the other hand, H-aggregates are characterized by the head-to-head packing of the transition dipoles, what results in the blue-shifted absorption and poor luminescence properties. Squaraines are known to form both types of aggregates depending on their structure and the environmental conditions^1^. The lack of systematic study of the self-assemblies and random reports of the J- or H-aggregation for various squaraines does provide only scarce information on the nature of the species present in the investigated case. Taking into account the numerous possible organization patterns in Langmuir–Blodgett films or virtually infinite number of oligomers and larger aggregates in solution, the precise control of the properties of the synthesized material is extremely hard. Therefore, the systematic investigation of the nature of the mutual interactions among the squaraine molecules first in vacuum and later on in various media is required.

The first step for the investigation of the self-aggregation is a careful analysis of the mutual interaction in the squaraine dimers. These issues so far have been analyzed only roughly, not systematically and sparse data are available in the literature. Although some crystalographic structures are available for squaraines, they do not contribute much to the data on the aggregation in solution. The theoretical analysis of the crystalographic structures and the discussion of the H-bonding pattern in crystals are mostly performed for dimers only^2,3^. Though nowadays the novel density functionals (DF) involving the dispersion correction are widely available and implemented in most popular quantum chemistry packages, still the articles appear in the literature that rely on the aggregate geometries optimized with conventional DF with no dispersion^2–4^. However, although proper for an isolated species, this methodology does not ensure the appropriate description of the weak van der Waals interaction that play an important role in the squaraine self-aggregation phenomena and thus leads to the structures that are artefacts of the applied approximation. Nevertheless, it fits to the described problem—the characteristics of the hydrogen bonding pattern. On the other hand, the density functional theory lacking the dispersion correction would not allow for the adequate inclusion of the mutual balance between the hydrogen bonding and $\pi$–$\pi$ attraction. Additionally also the size and flexibility of the basis set can have a crucial influence on the obtained optimized structures.

Thus the inclusion of the dispersion correction is mandatory for the density functional theory calculations of squaraine aggregates. Among the numerous available approaches to the dispersion correction for density functionals^5^, the highest popularity seems to be achieved by the Grimme’s atomistic a posteriori correction scheme. Beside the several variants of this correction, also the many-parameter functionals by Truhlar have been devised for noncovalent interactions^6,7^. In this case the inclusion of the kinetic energy density in both correlation and exchange parts of the functional is believed to improve the overall functional performance together with the elimination of the self-correlation error. It has been shown that particularly M05-2X with a double amount of non-local exchange gives the excellent performance for the weak interaction of the systems from the S22 test set both for the hydrogen-bonded and for dispersion-governed complexes^8^. Furthermore, the reoptimization of the long-range corrected functionals supplemented with the empirical dispersion correction proposed by Chai and Head–Gordon^9,10^ and denoted as $\omega \hbox {B97X}$-D delivers yet another option for the characterization of the noncovalent interactions. Moreover, the double-hybrids such as B2PLYP and B2PLYP-D^11,12^ have been proposed to improve the description of the electron correlation effects. The combination of a few ways of DF improving has lead to the reparametrized double-hybrids with an spin-component scalling and dispersion correction proposed by Martin et al.^13,14^. Though, despite the various dumping factors applied, empirical dispersion corrections suffer from the problems with the description of the dispersion contribution with an equal accuracy for the whole distance range. Thus, the new approach has been proposed based on the optimization of the DF parameters on the interaction energy with the dispersion component removed leading to the so called dispersionless DF (dlDF)^15^. Later on the dispersion energy is added computed in any adequate method (for instance SAPT in an implementation applied currently). The extensive reviews of the quality of the results for noncovalent interaction energies shows that among the best approximations B3LYP-D3, B97-D2, B97-D3 and B2PLYP-D are frequently referenced^16^. Thus, the aim of the present study is to provide the evaluation of the performance of the chosen popular density functionals and hybrid B2LYP method with various dispersion correction schemes for the description of the intermolecular interactions in 2,4-bis[4-(dimethyloamine)fenyl]cyclobutane-1,3-diole (DMASQ) dimers. The estimation of the quality of the obtained data is performed by the comparison with the MP2 level results. Additionally, the investigation of the nature of the mutual interactions in the dimers of various structure is performed with the (spin-component scaled) density-fitting zeroth-order symetry-adapted perturbation theory (SCS-)SAPT0^17,18^. One of the known shortcomings of this simplistic perturbational treatment that can prevail its advantages for some specific cases is the truncation of the intramonomer dispersion terms. Thus, in order to establish a correct energetic sequence in the analyzed systems of various nature (some with dominant dispersion forces and others governed mainly by electrostatic of hydrogen-bond contacts), MP2-coupled (MP2C) dispersion correction scheme is also applied and accounts for the reference level in the present case^19–21^.

## Technical details

Two different initial structures have been investigated: stacked (S) and hydrogen-bonded (Hb). According to the preliminary study, the two values of the monomer displacement were chosen for each type: short (s) and long (l). This allowed to generate four initial structures denoted as sS, lS, sHb and lHb. For all these structures the full geometry optimization was performed within the DFT approach.

Two popular functionals: B3LYP and B97 have been tested. In order to adequately describe the weak van der Waals interactions, the different Grimme’s dispersion correction schemes were applied: GD2, GD3 and GD3 with Becke–Johnson dumping (GD3BJ). For the geometry optimization, the correlation-consistent double-zeta basis set of Dunning was applied. The tight convergence criteria and the ultrafine grid for the numerical integration were applied together with the calculation of the force constants in each optimization step. For the final interaction study the four dimers have been chosen: sS, lS, lHb and lHb-B3LYP—the first three arising from the geometry optimization within the B3LYP-D3/cc-pVDZ approach and the latter one obtained with no dispersion correction in order to compare the stacked complexed with the planar one with the domination of the electrostatic contribution arising from hydrogen bond.

The supermolecular interaction energy for the obtained dimers have been calculated with various DFT and MP2 approaches. Since the MP2 method is known for very bad performance for interaction energies of aromatic stacked rings, also the MP2C interaction energies were investigated. As it was noticed by Hesselmann^19–21^, MP2C fixes the main error in supermolecular MP2 interaction energy which is incorrect dispersion contribution, introducing the time-dependent DFT coupled dispersion energy $E_{disp}^{TDDFT}$ in place of implicitly included UCHF dispersion term $E_{disp}^{UCHF}$^19^:

1 $$\begin{aligned} E_{int}^{MP2C}=E_{int}^{MP2}-E_{disp}^{UCHF}+E_{disp}^{TDDFT}. \end{aligned}$$

Basis set superposition error was eliminated by the application of the counterpoise procedure.

The interaction energy decomposition has been performed via the symmetry-adapted perturbation theory in its SAPT0 variant that neglects the intramonomer correlation effects. SAPT0 allows for the following decomposition of the interaction energy:

2 $$\begin{aligned} E_{SAPT0}=E_{elst}^{(10)}+E_{exch}^{(10)}+E_{ind,r}^{(20)}+E_{exch-ind,r}^{(20)}+E_{disp}^{(20)}+E_{exch-disp}^{(20)}, \end{aligned}$$

where the definitions of the subsequent terms can be found in the original works by Jeziorski, Moszynski and Szalewicz and in the review by Hohenstein and Sherrill^17,22^. It should be noticed that the zero in the acronym (SAPT0) and the second number in parentheses in the superscript of all terms denotes the order with respect to the intramonomer correlation operator. Such a simplification usually leads to the agreement with the benchmark values up to 20–30%^18^.

The geometry optimizations have been performed with Gaussian09^23^, while MP2C supermolecular energy calculations have been carried out in Molpro 2018.1^24,25^ and for all the remaining calculations including (SCS-)SAPT0 Psi4^26^ have been applied.

## Results and discussions

### Dimer structures

The geometry optimization has been performed for four initial structures: long displacement stacked, short displacement stacked, long displacement planar hydrogen bonded and short displacement hydrogen bonded. Depending on the approach applied for the geometry optimization, these four initial structures converge to different minima. However for all the approaches, several general observation can be wrought.

**Figure 2:**
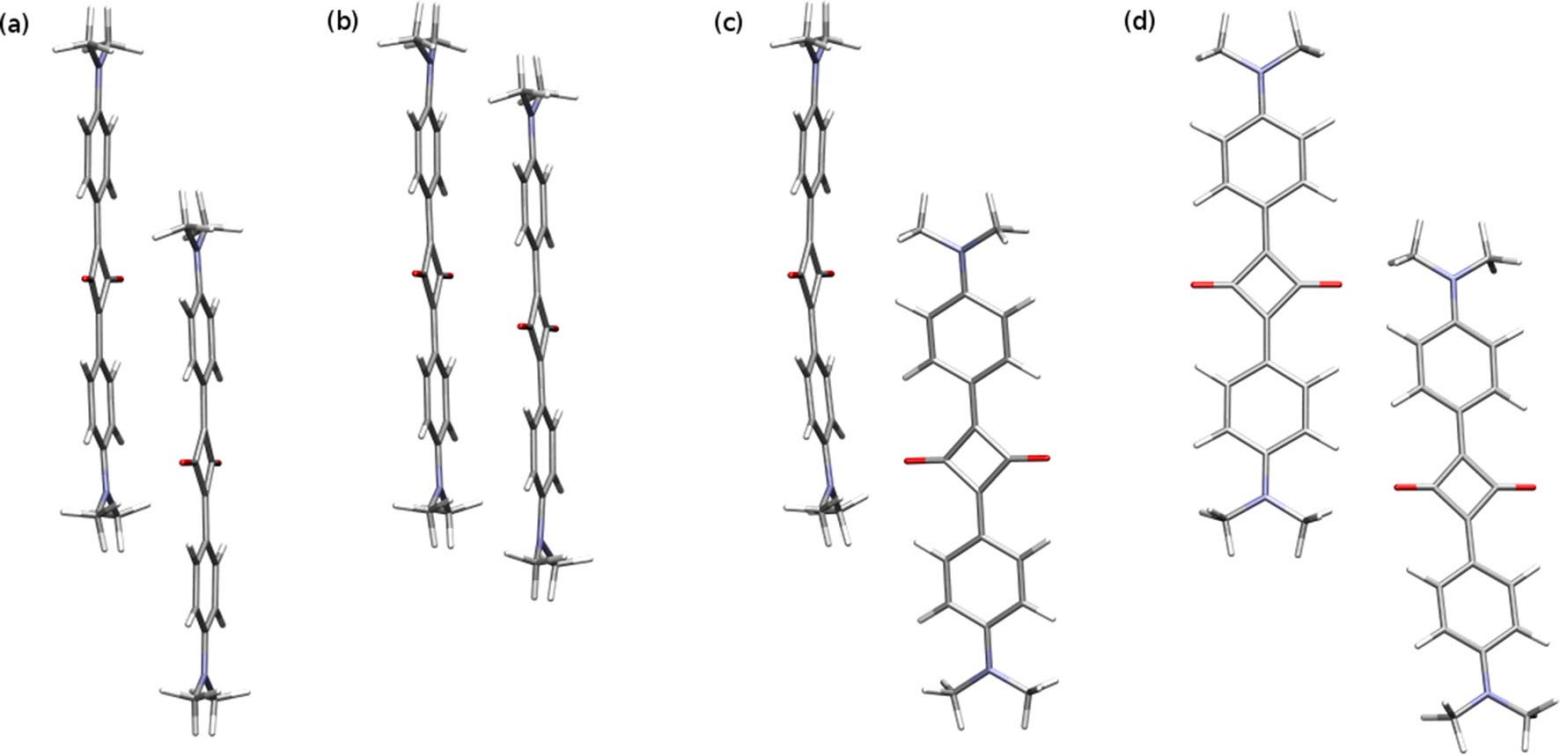
DMASQ dimer structures optimized with the B3LYP-D3/cc-pVDZ approach: (**a**) long displacement stacked (lS), (**b**) short displacement stacked (sS), (**c**) long hydrogen-bonded (lHb), and B3LYP/cc-pVDZ approach, (**d**) long hydrogen-bonded optimized with no dispersion included (lHb-B3LYP).

The geometry optimization with any method including the dispersion correction for the stacked dimers with both short and long displacement gives virtually the same optimized structures and the total energy difference of the order of 8–9 kcal/mol favouring the long displacement (Fig. 2, structure (a) favoured over (b)).

Starting geometry optimization from the H-bonded initial structure complicates the picture to the high extend. This arises from the fact that the potential energy surface for these two-body interactions is very complex and a high number of local minima. Thus, even such a delicate change of the computational methodology as the modification of the dispersion correction can lead to the localization of the different minima than those found in other approaches. This is the evidence that the proposed initial H-bonded structures lie far from the sought minima, as opposed to the case of the stacked dimers. Additionally, when applying the dispersion-corrected method, the initial H-bonded structures do converge to the stacked or perpendicular dimers (Fig. 2, structure (c)). Hence, in order to compare the dispersion-bound complexes with the H-bonded one, one dimer structure optimized within the conventional B3LYP functional with no dispersion (Fig. 2, structure (d)) need to be also included into the analysis.

Although the optimal geometries seem to be sensitive on the method of optimisation and initial structure, yet, they are all rather qualitatively similar. It is important to better understand the nature of intermolecular forces in DMASQ. In particular in next sections we explore the balance between dispersion and electrostatic interactions and assess the dispersion-corrected density functionals. These studies will focus on geometries presented in Fig. 2 .

### Interaction energy from symmetry-adapted perturbation theory

The influence of the basis set size on the interaction energies was investigated within the symmetry-adapted perturbation theory in its SAPT0 variant. Such a choice of the approximation was driven by its affordability even for relatively large systems. According to the recommendations by Hohenstein et al., the SAPT0 calculations in the small basis sets benefit from the error cancellation^17,18^. The remarkably good performance of SAPT0 was observed for correlation-consistent double-zeta basis sets with augmented functions restricted to up to p functions on heavy atoms (denoted by Hohenstein et al. as aug-cc-pVDZ’ and by Truhlar and coworkers and herein as jun-cc-pVDZ^27^). This combination of the approach and basis set is therefore endorsed as sufficient “for a semiquantitative analysis of the energy components”^18,28^. Additionally the test calculations performed by Wang et al.^29^ for the interaction of graphene with aromatic molecules reveal that indeed the SAPT0 makes best combination with the aug-cc-pVDZ basis set^29^.

**Figure 3:**
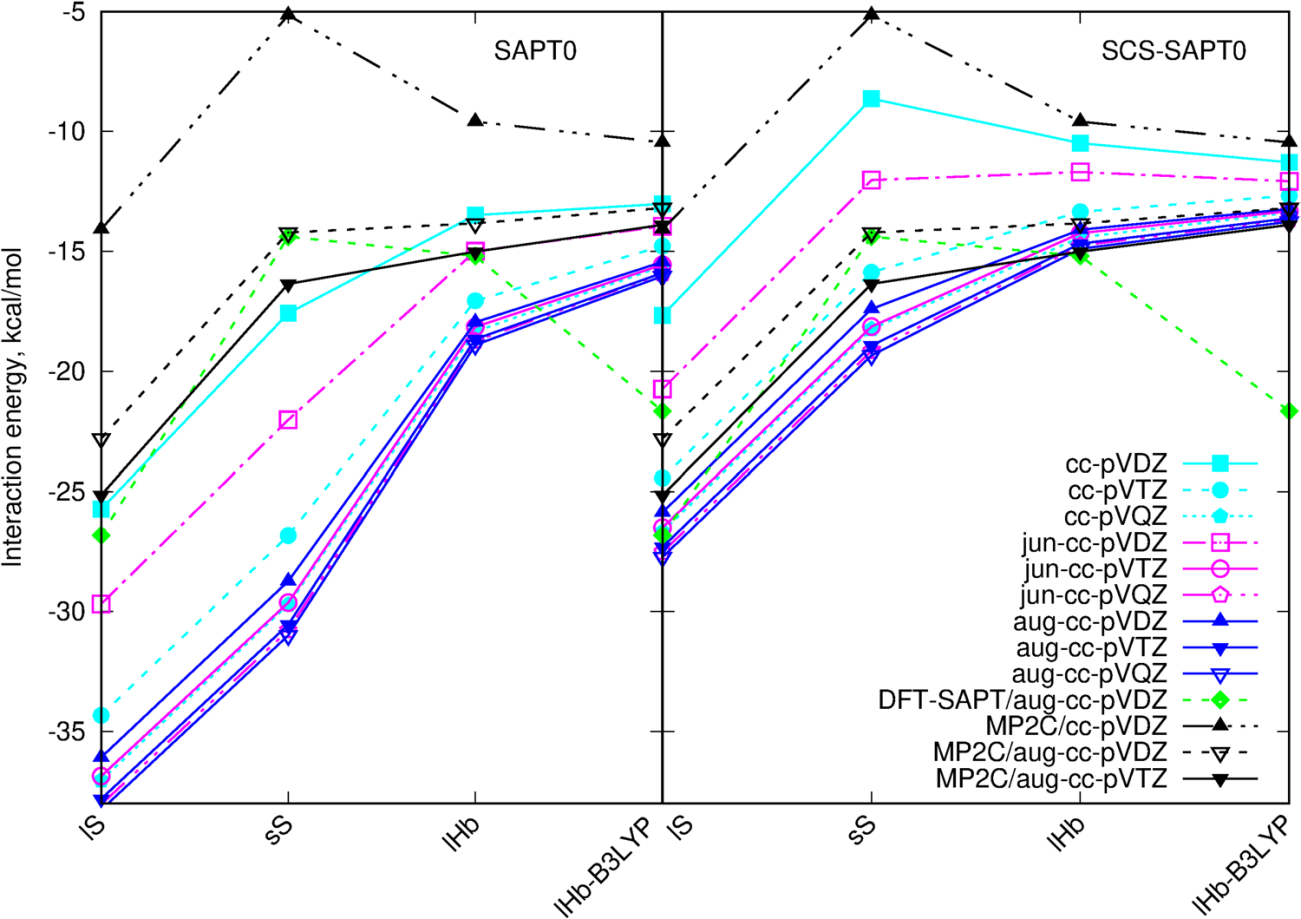
SAPT0 and SCS-SAPT0 interaction energy with various basis sets for the investigated DMASQ dimers compared with DFT-SAPT/aug-cc-pVDZ and MP2C total interaction energies.

Taking into account all the above mentioned recommendations, among the given results the SCS-SAPT0/aug-cc-pVDZ interaction energies can be taken as the best estimate. The total interaction energies for the investigated squaraine dimers are presented in Table 1 and Fig. 3. Independently of the basis set choice, the structure ordering with respect to the total SCS-SAPT0 interaction energy is the following: the strongest attraction is observed for the lS structure, and next sS, lHb and lHb-B3LYP take the place. The inclusion of the spin-component scalling significantly flattens the total interaction energy plot with respect to plain SAPT0 data, decreasing the relative energy differences between the different dimers. For the SCS-SAPT0/jun-cc-pVDZ approach, the strong attraction decrease (with respect to the SAPT0 with no SCS) is noted for the sS structure and all the interaction energies except the lS dimer become virtually the same and lie in the gap of 1 kcal/mol. SAPT0 without the spin-component scalling overestimates the total interaction energy by about 30% independently on the basis set, thus will be omitted in the further considerations.

The general analysis of the relative errors of the interaction energy with respect to the recommended SCS-SAPT0/aug-cc-pVDZ data reveals that among the spin-component corrected approaches the augmentation of the basis set with the diffuse functions leads to the differences smaller than 10%. The only exception is the jun-cc-pVDZ basis that is not sufficient even for the qualitative analysis. Due to the large size of the investigated systems and the convergence problems with the diffuse basis sets also the non-augmented basis sets can be considered, however particular care need to be taken. The present study indicates that cc-pVQZ basis set should be relatively safe, yet it is almost twice as big as jun-cc-pVTZ and more than three times as big as aug-cc-pVDZ (see Table 1). On the other hand cc-pVTZ with no diffuse functions though acceptable in size and good convergence, produces the errors as large as 10–15% for the dispersion-bound dimers. However, it is able to reproduce the correct energy ordering and thus can be applied conditionally in problematic cases when no quantitative conclusions need to be drawn. Additionally, it should be noticed that the small basis sets change the energetic sequence of the dimers: cc-pVDZ and jun-cc-pVDZ destabilize the sS structure artificially, hence confirming that the dispersion component of the interaction energy is the most sensitive on the basis set quality.

On the other hand, DFT-SAPT/aug-cc-pVDZ approach clearly favours lS dimer (by 5.16 kcal/mol), similarly as it is noticed in the case of SCS-SAPT0 approach, however the second stable dimer is the H-bonded (almost planar) structure from B3LYP optimization (lHb-B3LYP). It becomes by 6.47 kcal/mol more stable than perpendicular lHb dimer and by 7.27 kcal/mol more stable than sS structure. Such inversion of the energetic sequence against sS stacked structure with small monomer displacement arises mostly from the underestimation of electrostatic interaction in this dimer as well as in general problems with total dispersion component that becomes most significantly overestimated for hydrogen-bonded coplanar dimer lHb-B3LYP.

Due to the unconclusive results considering the SAPT0 dimer stability ordering, the MP2C data are added as a reference to comparison in Fig. 3. One can see that the basis set lacking any diffuse functions mimics the tendencies produced by DFT-SAPT: the weakest interaction is noticed for sS stacking dimer and second in line is lHb system. However an augmentation of the basis set with diffuse functions brings back the dimers ordering observed in SCS-SAPT0 approach, still the interaction energy differences between sS, lHb and lHb-B3LYP dimers become smaller than 2.5 kcal/mol. Therefore, MP2C approach with a diffuse basis set aug-cc-pVTZ would be taken here as the total reference ordering.

**Table 1:** Total SAPT0 and SCS-SAPT0 interaction energies for the dimer structures optimized within the cc-pVDZ basis set (B3LYP-D3 functional unless stated otherwise) (kcal/mol).

Dimer	#bf	lS	sS	lHb	lHb-B3LYP
**SAPT0**					
cc-pVDZ	872	$-$25.74	$-$ 17.58	$-$ 13.49	$-$ 13.02
cc-pVTZ	2000	$-$ 34.33	$-$ 26.83	$-$ 17.06	$-$ 14.78
cc-pVQZ	4760	$-$ 37.04	$-$ 29.71	$-$ 18.34	$-$ 15.62
jun-cc-pVDZ	1064	$-$ 29.68	$-$ 22.03	$-$ 15.00	$-$ 13.95
jun-cc-pVTZ	2432	$-$ 36.86	$-$ 29.63	$-$ 18.15	$-$ 15.56
jun-cc-pVQZ	4608	$-$ 38.04	$-$ 30.78	$-$ 18.74	$-$ 15.93
aug-cc-pVDZ	1464	$-$ 36.08	$-$ 28.74	$-$ 17.96	$-$ 15.44
aug-cc-pVTZ	3128	$-$ 37.81	$-$ 30.55	$-$ 18.66	$-$15.90
aug-cc-pVQZ	5680	$-$ 38.27	$-$ 30.99	$-$ 18.90	$-$ 16.04
**SCS-SAPT0**					
cc-pVDZ	872	$-$ 17.67	$-$ 8.63	$-$ 10.49	$-$ 11.29
cc-pVTZ	2000	$-$ 24.44	$-$ 15.87	$-$ 13.35	$-$ 12.68
cc-pVQZ	4760	$-$ 26.67	$-$ 18.24	$-$ 14.42	$-$ 13.39
jun-cc-pVDZ	1064	$-$ 20.74	$-$ 12.03	$-$ 11.69	$-$ 12.08
jun-cc-pVTZ	2432	$-$ 26.50	$-$ 18.14	$-$ 14.24	$-$ 13.33
jun-cc-pVQZ	4608	$-$ 27.52	$-$ 19.15	$-$ 14.76	$-$ 13.66
aug-cc-pVDZ	1464	$-$ 25.86	$-$ 17.40	$-$ 14.10	$-$ 13.23
aug-cc-pVTZ	3128	$-$ 27.31	$-$ 18.93	$-$ 14.68	$-$ 13.62
aug-cc-pVQZ	5680	$-$ 27.73	$-$ 19.34	$-$ 14.90	$-$ 13.76
**DFT-SAPT**					
aug-cc-pVDZ	1464	$-$ 26.82	$-$ 14.39	$-$ 15.19	$-$ 21.66
**MP2C**					
cc-pVDZ	872	$-$ 14.08	$-$ 5.16	$-$ 9.59	$-$ 10.46
aug-cc-pVDZ	1464	$-$ 22.80	$-$ 14.22	$-$ 13.83	$-$ 13.19
aug-cc-pVTZ	3128	$-$ 25.15	$-$ 16.36	$-$ 15.01	$-$ 13.90

#bf denotes number of basis functions. For comparison also the DFT-SAPT/aug-cc-pVDZ and MP2C results are presented.

### Interaction energy decomposition: SCS-SAPT0

**Figure 4:**
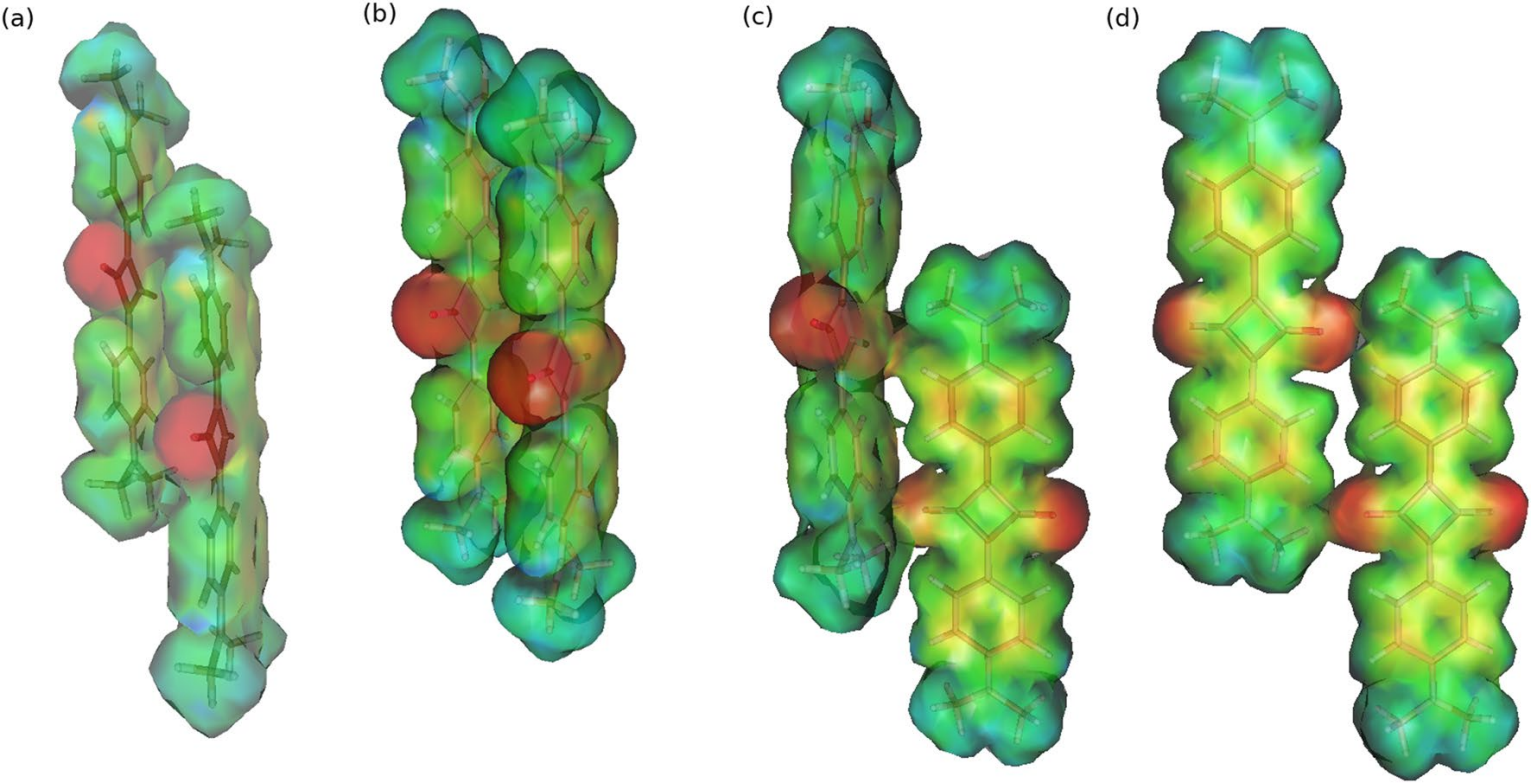
Multipole-derived electrostatic potential of DMASQ dimers optimized with the B3LYP-D3/cc-pVDZ approach: (**a**) lS, (**b**) sS, (**c**) lHb, and B3LYP/cc-pVDZ approach (**d**) lHb-B3LYP.

The interaction energy components obtained with SCS-SAPT0 procedure are presented in Table 2 and for comparison also a visualization of multipole-derived electrostatic potential is given in Fig. 4. Considering the dispersion-bound complexes as these for which the dispersion energy contribution is at least two times larger than electrostatics, the dispersion character can be ascribed for the two among the investigated dimers: lS and sS (see Table 2). The highest influence of the dispersion contribution is noticed for the sS system, when the short relative translation of the monomers leads to direct interaction of the aromatic rings of two subsystems as in paralel-displaced benzene dimer. On the other hand, in lS dimer the larger translation of the subsystems causes the interaction (of mostly electrostatic nature) of the squaric ring of the partially positive charge from the one molecule with the electron rich nitrogen of amino group from the other thus slightly diminishing the importance of the dispersion interaction (compare Fig. 4). The remaining two dimers with the dispersion of the order of 1.24 and 0.87 electrostatic contribution respectively for lHb and lHB-B3LYP with aug-cc-pVDZ basis set, possess the mixed character.

**Figure 5:**
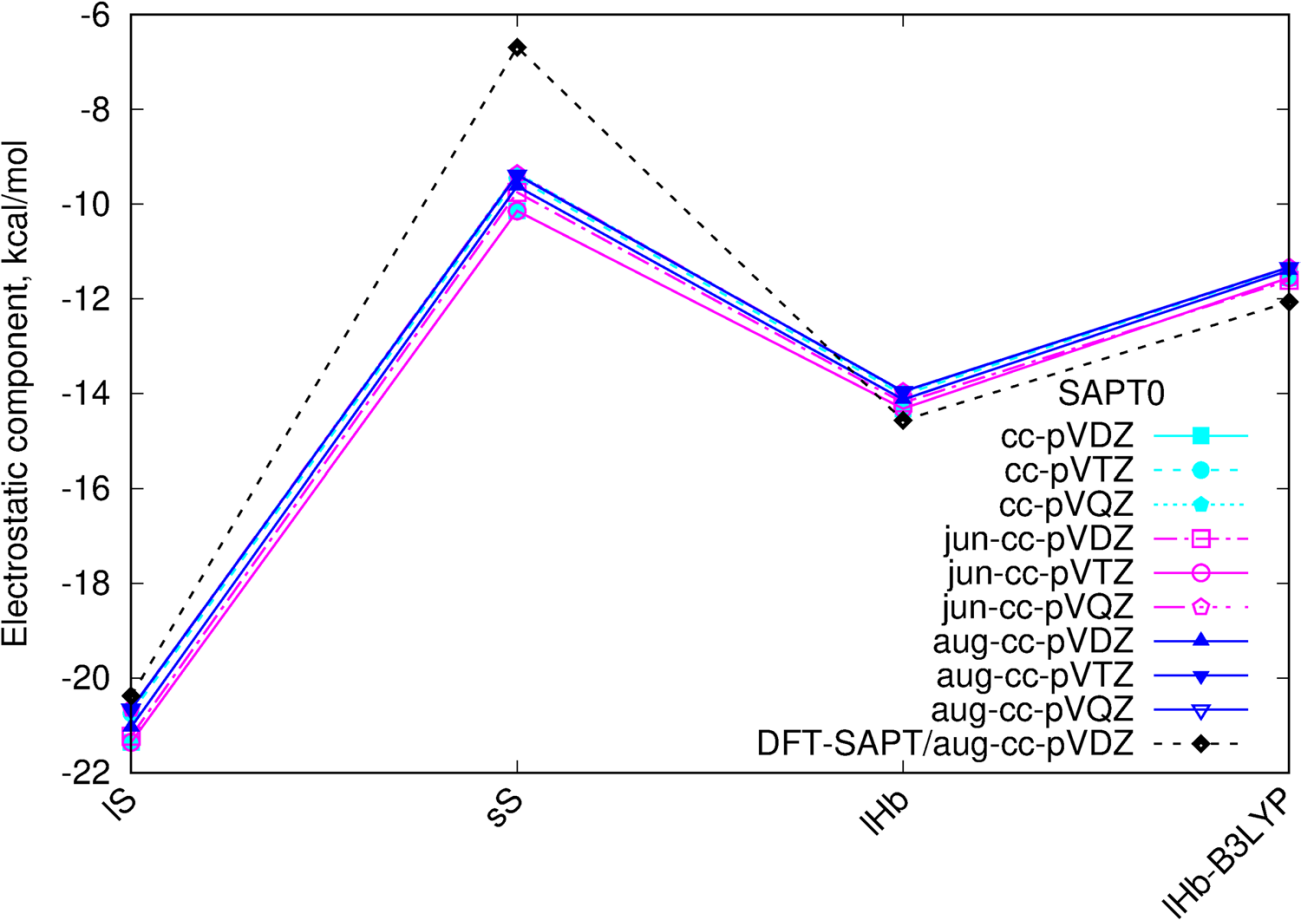
Electrostatic component of the SAPT0 interaction energy with various basis sets. The same linetype and point shape for the same basis set on both panels.

Electrostatic component of the total interaction energy is not sensitive for a basis set choice. The consistent results for all dimers in SAPT0 decompositions are obtained even in the smallest applied cc-pVDZ basis set with no diffusion functions (Fig. 5).

**Figure 6:**
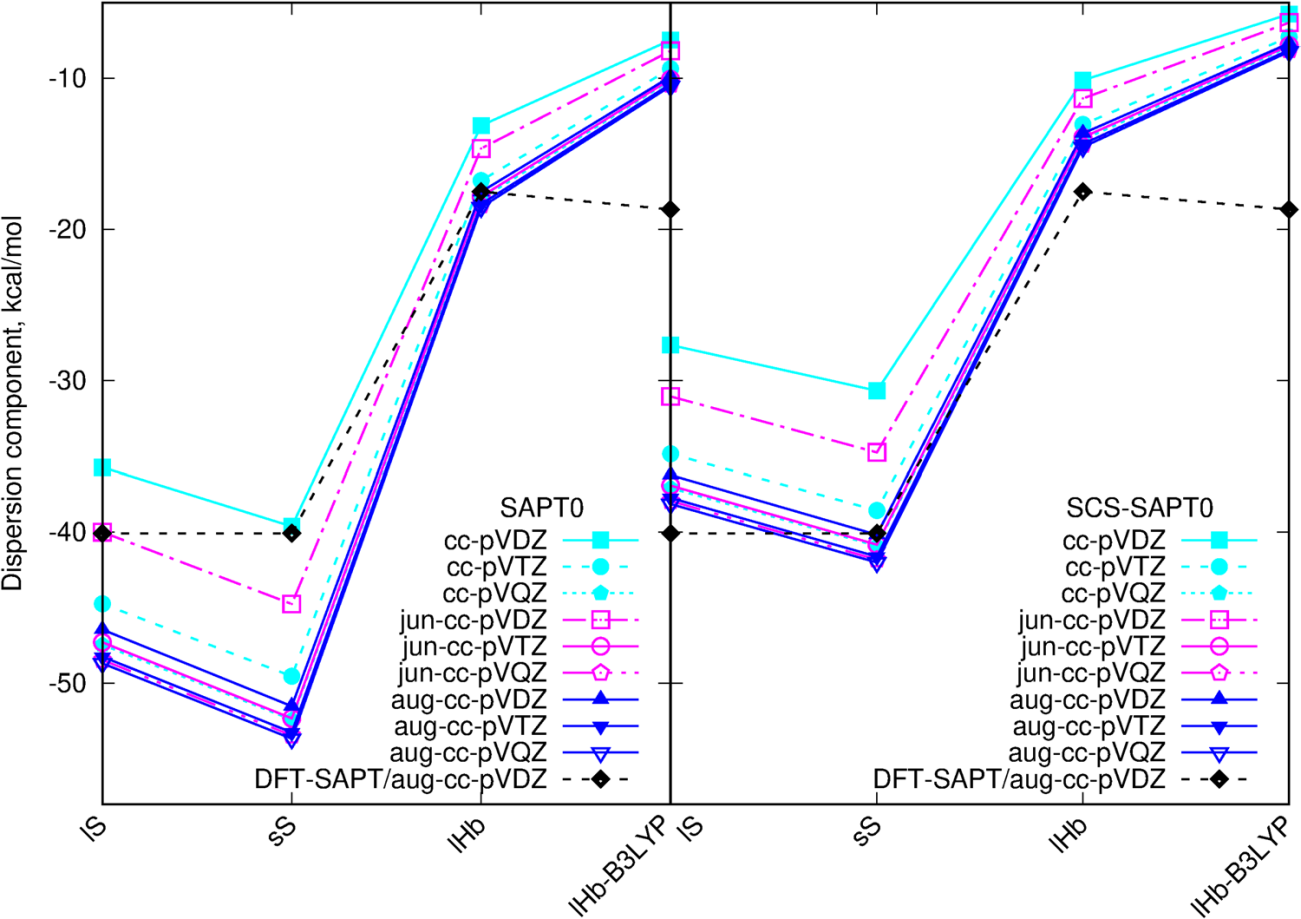
Dispersion component of the SAPT0 interaction energy with various basis sets. The same linetype and point shape for the same basis set on both panels.

On the other hand and in agreement with a general knowledge all the data for the dispersion energy component suggest that in the case of the absence of the diffuse functions, the basis set of at least triple-zeta quality should be applied. The dispersion energy components obtained with different basis sets are presented in Table 3 and in Fig. 6. The double-zeta basis set with jun-augmentation produce the relative errors with respect to the aug-cc-pVDZ data larger than 10%. The double-zeta basis void of diffuse functions leads to the relative errors larger than 20%. In this manner, both cc-pVDZ and jun-cc-pVDZ can distort even the qualitative considerations and cannot be recommended. For the basis sets of the triple-zeta quality and larger, the amount of the included diffuse functions have minor influence on the results quality—the reduced jun basis by Truhlar allows for the good reproduction of the fully augmented basis set data retaining the reasonable cost of calculations.

In the light of the above analysis, the (SCS-)SAPT0 calculations with jun basis sets of the triple-zeta quality can be recommended in the cases when only semiquantitative considerations are carried on. In the present case its errors do not exceed 2% with respect to the reference SAPT0/aug-cc-pVDZ data. Moreover, for the qualitative analysis also the triple-zeta basis sets with no diffuse functions can be applied giving the satisfactory accuracy (of the order of 5%), however they need to be employed with care. These observations are of particular importance for the calculations for the large molecular systems, where the inclusion of the diffusion functions to the basis set significantly increases the time and resources necessary for calculations and additionally can cause the tremendous problems with the SCF convergence.

One can also notice that in contrast to (SCS-)SAPT0, for DFT-SAPT the dispersion energy component presented in Table 3 is almost identical for lS and sS squaraine dimer and close to each other for both Hb systems. This overestimation of the stability of the long-displaced stacked dimer (lS) and lHB-B3LYP one leads to the disturbed total interaction energy ordering for DFT-SAPT (Fig. 3) with respect to the stability estimated with MP2C or SAPT0 approaches.

**Table 2:** (SCS-)SAPT0 interaction energy components for the dimer structures in aug-cc-pVDZ and aug-cc-pVQZ (kcal/mol).

Dimer	aug-cc-pVDZ				aug-cc-pVQZ			
	lS	sS	lHb	lHb-B3LYP	lS	sS	lHb	lHb-B3LYP
Electrostatics	$-$ 21.04	$-$ 9.62	$-$ 14.13	$-$ 11.40	$-$ 20.65	$-$ 9.39	$-$ 13.96	$-$ 11.34
$\hbox {E}_{elst,r}^{(10)}$	$-$ 21.04	$-$ 9.62	$-$ 14.13	$-$ 11.40	$-$ 20.65	$-$ 9.39	$-$ 13.96	$-$ 11.34
Exchange	37.67	36.90	19.60	11.09	37.32	36.55	19.54	11.08
$\hbox {E}_{exch}^{(10)}$	37.67	36.90	19.60	11.09	37.32	36.55	19.54	11.08
$\hbox {E}_{exch}^{(10)}(\hbox {S}{^2})$	37.66	36.88	19.52	11.06	37.31	36.52	19.46	11.04
Induction	$-$ 6.26	$-$ 4.51	$-$ 5.91	$-$ 5.22	$-$ 6.25	$-$4.49	$-$5.93	$-$5.25
$\hbox {E}_{ind,r}^{(20)}$	$-$ 16.21	$-$ 15.29	$-$ 7.92	$-$ 5.20	$-$ 16.73	$-$ 15.72	$-$ 8.21	$-$ 5.39
$\hbox {E}_{exch-ind,r}^{(20)}$	13.50	13.51	4.71	1.78	14.04	13.98	5.00	1.95
$\delta ^{HF}_{r}(2)$	$-$ 3.55	$-$ 2.73	$-$ 2.70	$-$ 1.79	$-$ 3.56	$-$ 2.74	$-$ 2.72	$-$ 1.81
Dispersion	$-$ 46.45	$-$ 51.51	$-$ 17.52	$-$ 9.90	$-$ 48.68	$-$ 53.66	$-$ 18.55	$-$ 10.53
$\hbox {E}_{disp}^{(20)}$	$-$ 52.30	$-$ 57.63	$-$ 19.68	$-$ 10.98	$-$ 55.02	$-$ 60.24	$-$ 20.97	$-$ 11.76
$\hbox {E}_{exch-disp}^{(20)}$	5.85	6.12	2.16	1.07	6.33	6.57	2.42	1.23
SCS Dispersion	$-$ 36.23	$-$ 40.17	$-$ 13.66	$-$ 7.70	$-$ 38.14	$-$ 42.01	$-$ 14.56	$-$ 8.25
$\hbox {E}_{disp}^{(20)}\hbox {(SS)}$	$-$ 26.15	$-$ 28.81	$-$ 9.84	$-$ 5.49	$-$ 27.51	$-$ 30.12	$-$ 10.48	$-$ 5.88
$\hbox {E}_{disp}^{(20)}\hbox {(OS)}$	$-$ 26.15	$-$ 28.81	$-$9.84	$-$5.49	$-$27.51	$-$ 30.12	$-$ 10.48	$-$5.88
$\hbox {E}_{exch-disp}^{(20)}\hbox {(SS)}$	3.64	3.84	1.34	0.66	4.12	4.29	1.59	0.82
$\hbox {E}_{exch-disp}^{(20)}\hbox {(OS)}$	2.21	2.28	0.82	0.41	2.22	2.28	0.82	0.41
Total HF	10.37	22.77	$-$ 0.44	$-$ 5.53	10.41	22.67	$-$ 0.35	$-$ 5.51
Total SAPT0	$-$ 36.08	$-$ 28.74	$-$ 17.96	$-$ 15.44	$-$ 38.27	$-$30.99	$-$ 18.90	$-$ 16.04
Total SCS$-$SAPT0	$-$ 25.86	$-$ 17.40	$-$ 14.10	$-$13.23	$-$ 27.73	$-$ 19.34	$-$ 14.90	$-$ 13.76
Disp/Elst ratio	2.21	5.35	1.24	0.87	2.36	5.71	1.33	0.93

**Table 3:** (SCS-)SAPT0 dispersion energy components for the dimer structures [kcal/mol] (#bf denotes number of basis functions).

Dimer	#bf	lS		sS		lHb		lHb-B3LYP	
Dispersion									
cc-pVDZ	872	$-$ 35.73	(23.08)	$-$ 39.62	(23.08)	$-$ 13.14	(25.00)	$-$ 7.49	(24.34)
cc-pVTZ	2000	$-$ 44.74	(3.68)	$-$ 49.53	(3.84)	$-$ 16.78	(4.22)	$-$ 9.38	(5.25)
cc-pVQZ	4760	$-$ 47.45	($-$ 2.15)	$-$ 52.40	($-$ 1.73)	$-$ 18.00	($-$ 2.74)	$-$ 10.15	($-$ 2.53)
jun-cc-pVDZ	1064	$-$ 40.00	(13.89)	$-$ 44.76	(13.10)	$-$ 14.66	(16.32)	$-$ 8.20	(17.17)
jun-cc-pVTZ	2432	$-$ 47.29	($-$ 1.81)	$-$ 52.31	($-$ 1.55)	$-$ 17.83	($-$ 1.77)	$-$ 10.08	($-$ 1.82)
jun-cc-pVQZ	4608	$-$ 48.45	($-$ 4.31)	$-$ 53.45	($-$ 3.77)	$-$ 18.40	($-$ 5.02)	$-$ 10.43	($-$ 5.35)
aug-cc-pVDZ	1464	$-$ 46.45	(0.00)	$-$ 51.51	(0.00)	$-$ 17.52	(0.00)	$-$ 9.90	(0.00)
aug-cc-pVTZ	3128	$-$ 48.24	($-$ 3.85)	$-$ 53.24	($-$ 3.36)	$-$ 18.34	($-$ 4.68)	$-$ 10.40	($-$ 5.05)
aug-cc-pVQZ	5680	$-$ 48.68	($-$ 4.80)	$-$ 53.66	($-$ 4.17)	$-$ 18.55	($-$ 5.88)	$-$ 10.53	($-$ 6.36)
SCS-Dispersion									
cc-pVDZ	872	$-$ 27.66	(23.65)	$-$ 30.68	(23.62)	$-$ 10.15	(25.70)	$-$ 5.76	(25.19)
cc-pVTZ	2000	$-$ 34.85	( 3.81)	$-$ 38.57	( 3.98)	$-$ 13.07	( 4.32)	$-$ 7.28	( 5.45)
cc-pVQZ	4760	$-$37.09	($-$2.37)	$-$40.92	($-$1.87)	$-$14.08	($-$3.07)	$-$7.92	($-$2.86)
jun-cc-pVDZ	1064	$-$31.06	(14.27)	$-$34.76	(13.47)	$-$11.35	(16.91)	$-$6.33	(17.79)
jun-cc-pVTZ	2432	$-$ 36.93	($-$ 1.93)	$-$ 40.83	($-$ 2.45)	$-$ 13.92	($-$ 1.90)	$-$ 7.85	($-$ 1.95)
jun-cc-pVQZ	4608	$-$ 37.94	($-$ 4.72)	$-$ 41.82	($-$ 4.10)	$-$ 14.42	($-$ 5.56)	$-$ 8.15	($-$ 5.84)
aug-cc-pVDZ	1464	$-$ 36.23	(0.00)	$-$ 40.17	(0.00)	$-$ 13.66	(0.00)	$-$ 7.70	( 0.00)
aug-cc-pVTZ	3128	$-$ 37.74	($-$ 4.17)	$-$ 41.62	($-$ 3.61)	$-$ 14.36	($-$ 5.12)	$-$ 8.13	($-$ 5.58)
aug-cc-pVQZ	5680	$-$ 38.14	($-$ 5.27)	$-$ 42.01	($-$ 4.58)	$-$ 14.56	($-$ 6.59)	$-$ 8.25	($-$ 7.14)
DFT-SAPT/aug-cc-pVDZ		− 40.09		− 40.07		− 17.51		− 18.68	

The relative errors in percent with respect to the (SCS-)SAPT0/aug-cc-pVDZ results are given in parenthesis. For comparison also the DFT-SAPT/aug-cc-pVDZ results are presented.

### Supermolecular interaction energy

**Figure 7:**
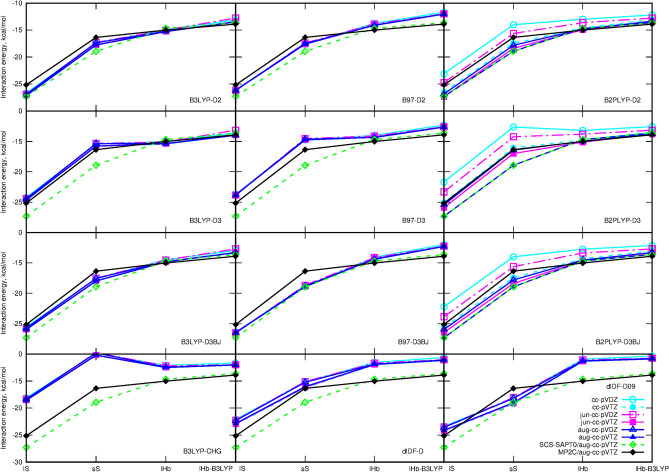
Supermolecular counterpoise-corrected interaction energy with various dispersion corrections compared to the SCS-SAPT0/aug-cc-pVTZ and MP2C/aug-cc-pVTZ data.

**Figure 8:**
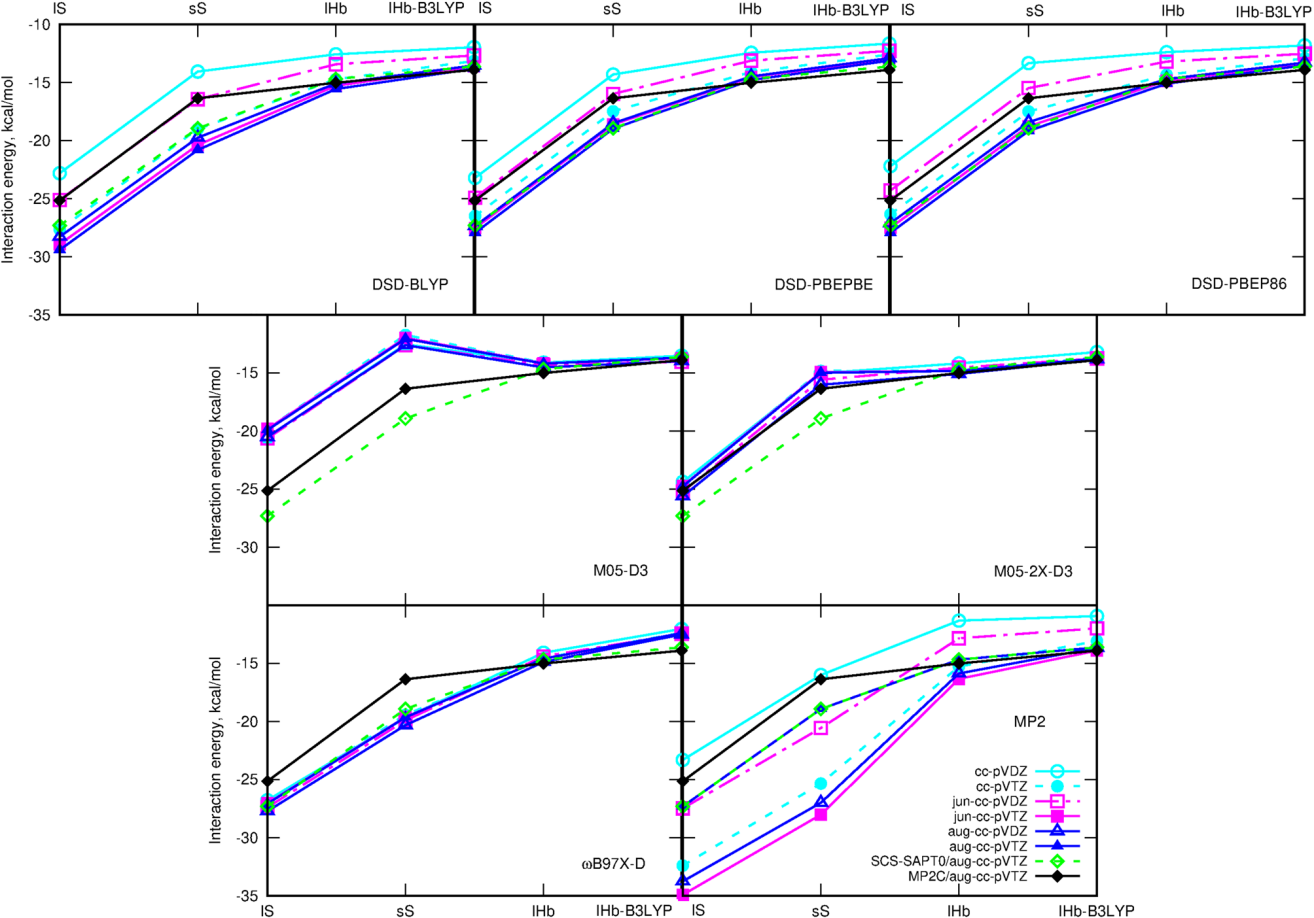
Supermolecular counterpoise-corrected interaction energy with DSD functionals and MP2 approach compared to the SCS-SAPT0/aug-cc-pVTZ and MP2C/aug-cc-pVTZ data.

The obtained supermolecular interaction energies for commonly used density functionals are plotted in Figs. 7 and 8. Additionally, Supporting Information contains tabelarized counterpoise-corrected results for all the investigated functionals and various basis sets.

It can be noticed that almost all applied approaches allow to reproduce the general tendencies observed for the analyzed squaraine dimers. Some tiny problems regarding the relative stability of sS and lHb dimers can be observed in the case of the D3 dispersion correction with the original D3 dumping (denoted here simply as D3) independently on the functional employed in calculations. Here, though the energetic ordering of the dimers can be preserved for most basis sets, the energy difference between sS and lHb dimers appears to be diminished to tenth parts of kcal/mol and the energy profiles in Fig. 7 become flatter than in the MP2C/aug-cc-pVTZ reference case. The energy difference between sS and lHB dimers in B3LYP-D3 approach does not exceed 0.43 kcal/mol and in most basis sets is significantly smaller (such as 0.02 kcal/mol for aug-cc-pVDZ). In the case of MP2C, the corresponding energy difference is equal to 1.35 kcal/mol for aug-cc-pVTZ basis set. Interestingly, these difficulties are observed only in the case of the dispersion bound sS dimers and for the side/hydrogen-bonded systems the D3 correction can be applied with the same effects as D2 or D3BJ ones. Similar remark can be carried out for the Chai–Head–Gordon dispersion correction with B3LYP functional—it significantly destabilizes the sS dispersion-governed dimers with respect to the hydrogen-bonded ones.

The particularly good correspondence of the supermolecular results with the SCS-SAPT0 and reference MP2C data together with the small basis set dependence of the obtained data can be noticed for B3LYP-D3 and M05-2X-D3 functionals with counterpoise correction.

Moreover, the data obtained with the GGA or hybrid functional are negligibly affected by the basis set applied. Even the small basis set not containing any diffuse functions allow to obtain the relative errors of the order of several precents—similarly as for the much larger aug-cc-pVDZ or aug-cc-pVTZ basis sets. However, this is not the case for the double-hybrid methods and MP2 approach. Here the small basis sets lacking the diffuse functions or including only d functions on heavy atoms (jun-type) magnify the errors.

Along with the observations for SAPT0 convergence with respect to the basis set choice, also for supermolecular interaction energy the problems appear mostly for the dispersion bound dimers, while for the H-bonded ones already jun-cc-pVDZ or cc-pVTZ reproduce MP2C/aug-cc-pVTZ with an accuracy acceptable for qualitative considerations independently on the functional chosen. Still, the application of any dispersion correction is mandatory for both types of dimers even for the double-hybrid functionals containing the MP2-like correlation contribution.

Since the Grimme dispersion correction was parametrized not taking into account the counterpoise correction for the weakly interacting complexes, it is recommended to apply it as parametrized. Therefore, the careful investigation of the uncorrected interaction energy values is also performed here for the squaraine dimers. Figures and tables in Supporting information summarize the obtained numbers. As could be expected, one can clearly see, that the data are affected by the basis set size to the significantly higher extend than in the case of the counterpoise corrected energies. Moreover, double hybrids (including DSD functionals) and MP2 significantly overestimate the dimer stabilization. Interestingly, in the case of these methods the worst correspondence of the uncorrected interaction energy to the reference MP2C/aug-cc-pVTZ data is obtained not for the smallest cc-pVDZ basis set, but for the aug-cc-pVDZ one despite the contained diffuse functions. This suggest that the aug-cc-pVDZ basis set should be avoided or at least applied with a particular care mainly in a second-order Møller–Plesset perturbation theory estimation of supermolecular interaction energy without the counterpoise correction. For B3LYP and B97 functionals with various dispersion correction, intuitively, best uncorrected interaction energies are obtained with the jun-cc-pVTZ and aug-cc-pVTZ basis sets. D3 correction favoures also the cc-pVTZ basis set with no diffuse functions, but this should be again treated as the fortitious cancellation of errors and not the superiority of this basis set.

The numerous publications devoted to the magnitude of the basis set superposition error in various approaches for the interaction energy calculations can be found in literature. Among others there are the reports on the large values of the basis set superposition error in the MP2 interaction energies^30–32^. It is also known that DFT functionals are usually less affected by BSSE than other methods^32–34^. Moreover, the presence of the MP2-like correlation admixture in the double-hybrid functionals increases their sensitivity to the BSSE up to the level observed in the MP2 calculations. However, the ongoing progress in the new methods development requires the continuous careful determination of the influence of the basis set inconsistencies for the calculated weak intermolecular interaction energies. Due to the parallel changes of the dispersion contribution to the interaction energy and BSSE, one can expect the most problematic situations for the dispersion-bound dimers.

The BSSE values obtained in the present study are presented in Supporting Information. An analysis of the percentage of BSSE with respect to the total supermolecular interaction energy determined with the same approach clearly reveals the poor quality of the non-corrected $\Delta E$ for any approximation containing the explicit perturbation correlation component (double hybrids and MP2). For all these cases the BSSE obtained with any basis set is larger than 10% of the total CP-corrected interaction energy. The remaining functionals: B3LYP, B97, $\omega \hbox {B97X}$-D, Minnesota functionals and dlDF applied with jun-cc-pVTZ or aug-cc-pVTZ basis sets reproduce the supermolecular interaction energy with the accuracy smaller or equal to 10%, what can be acceptable for qualitative considerations. The highest BSSE values are noticed for the system with the largest dispersion energy component, namely sS. Here even the triple-zeta quality basis set augmented with diffusion functions does not allow to minimize this error to the satisfactory level. On the other hand, the hydrogen-bonded lHb-B3LYP dimer in most cases suffers from the smallest BSSE value, except for the cc-pVDZ basis set.

### Squaraine interaction in context of model aromatic dimers and other aggregates

**Figure 9:**
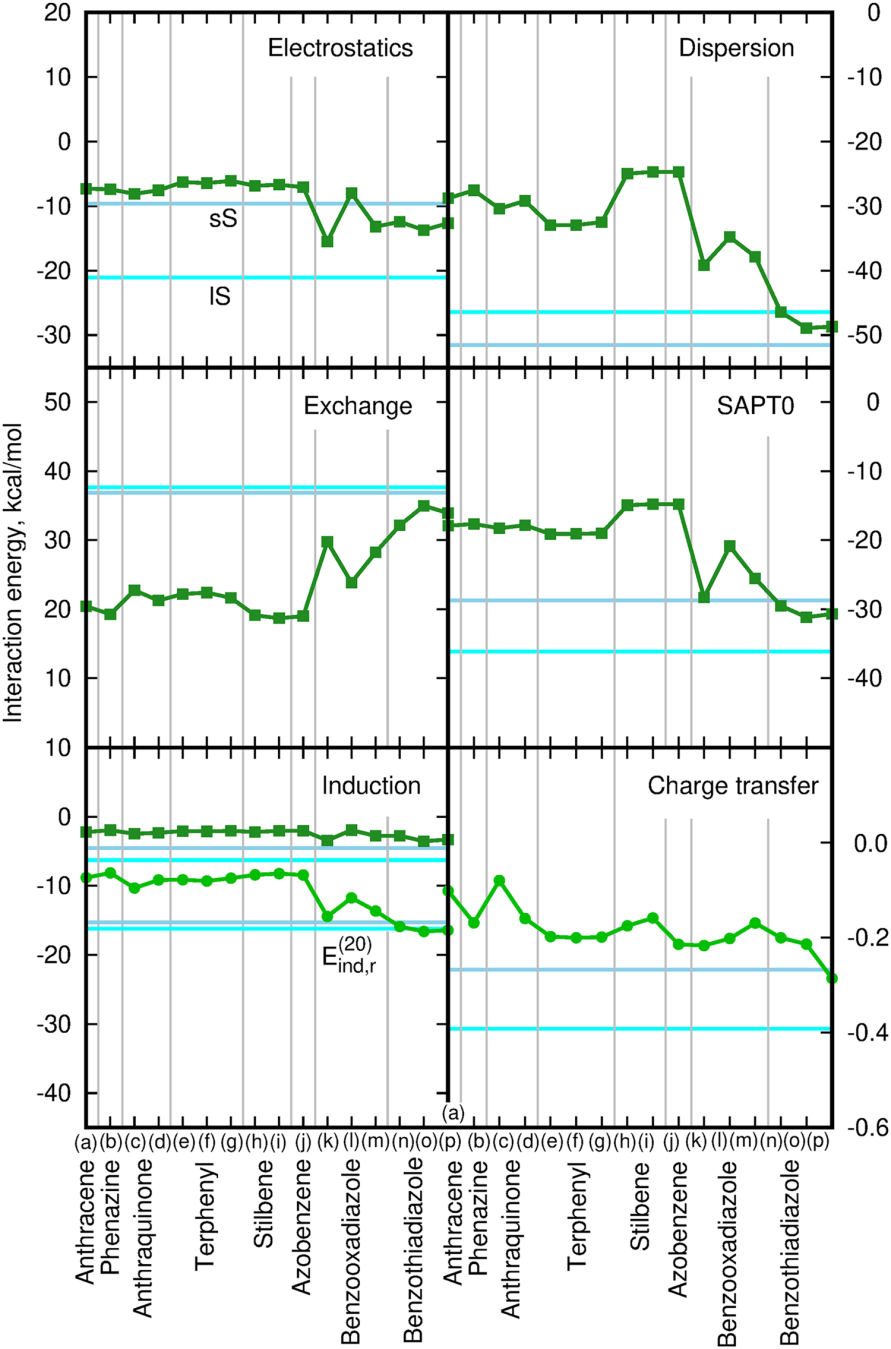
SAPT0 interaction energy components for selected cumulated and conjugated dimers with respect to the sS and lS squaraine dimers. The horizontal cyan line depicts the energy value for the lS squaraine dimer, the horizontal blue line refers to the sS squaraine dimer energy values. In the Induction panel the squares depict the total induction energy component and circles—the pure $E_{ind,r}^{(20)}$ component. Corresponding structures (a)–(p) and numerical values of SAPT0 interaction energy components provided in Suppporting Information. For clarity, all components except charge transfer are presented with the same range of y values.

In order to settle the squaraine interaction in context of polycyclic aromatic hydrocarbon interactions conventionally applied for testing of each new methodology and thus well described and other polar aromatic compounds containing heteroatoms, the SAPT0 interaction energy for all four analyzed squaraine dimers was matched against four groups of simple model systems. First of them contains cumulated aromatic rings and includes anthracene, phenazine and antrachinone dimers, while the second one consists of three simple conjugated molecules containing aromatic rings and optionally also heteroatoms, namely therphenyl, stilbene and azobenzene dimers. Additionaly, more complex donor–acceptor–donor species were also included into consideration, as resembling at best the quadrupolar character of squaraine molecules, namely benzoxadiazole and benzothiadiazole fluorinated derivative. The last class of model reference systems consists of the dimers classically considered in the aspect of self-aggregation: terthiophene dimers as the oligomers exhibiting H-aggregate formation and pseudoisocyanine chloride dimers initially investigated in the context of J-aggregation by Jelley and Scheibe^35–37^. The structures of all reference dimers are presented in Supporting Information in Fig. S1. It should be underlined that these are not the complete set of possible stable dimers, but only selected minima on the potential energy surface, optimized with the B3LYP-D3/cc-pVDZ approach, that can be directly compared with the lS and sS squaraine complexes from Fig. 1. Figure 9 presents the SAPT0/aug-cc-pVDZ interaction energy and its components for the three above mentioned groups. The horizontal cyan and light blue lines correspond to the interaction energy components for lS and sS squaraine dimers, respectively. For the simplicity of comparison all of the components except charge transfer contribution have been presented with the same range at the ordinate axis. One can see that for the analyzed squaraine dimers the electrostatic component of sS complex closely resemble the wide range of investigated dimers both of the cummulated and conjugated character. Similarly, the induction components of all of the analyzed dimers exhibit the qualitative similarity, either for the pure $E_{ind,r}^{(20)}$ term (light green line with circles) or for all the summed up induction-type contributions ($E_{ind,r}^{(20)}$, $E_{exch-ind,r}^{(20)}$ and $\delta _{HF,r}(2)$—dark green line with squares). The strength of the induction and dispersion interactions can be rationalized by inspection of the ionization potentials and vertical excitation energies, as they are roughly inversly proportional to them. Taking the qualitative calculations within a M06-2X/6-31+G(d,p) approach (proven relativly good for IP prediction^38^ among DFT functionals which in general totally fail in experimental IP values reproduction), the good correspondence has been obtained between the induction energy component and IP estimations (squaraine IP is predicted to be 6.45 while for the subsequent monomers: anthracene, phenazine, antraquinone, terphenyl, stilbene and azobenzene the corresponding values are equal respectively 7.42 eV, 8.38 eV, 9.77 eV, 7.93 eV, 7.69 eV and 8.69 eV). It is yet remarkable that the dispersion energy component is much stronger for squaraines than for any other considered aggregates except D-A-D model benzothiadiazole dimers. This makes the overall attraction of stacked molecules stronger despite the significant positive exchange component. However, according to the expectations, the general tendency for the dispersion component closely follows the values of the vertical excitation energy, since the expression for the dispersion energy contains the numerator with the transition moment integrals and denominator with the ground and excited state energy difference, $\Delta E_{Xk}=E_{Xk}-E_{X0}$ (where 0 denotes the ground state and *k* enumerates the excited states for subsystem *X*),

3 $$\begin{aligned} E_{disp}\sim -\sum \frac{|\langle \psi _{A0}\psi _{B0}|V|\psi _{Ai}\psi _{Bj} \rangle |^2}{\Delta E_{Ai}+\Delta E_{Bj}}. \end{aligned}$$

Therefore, one can assume that for the similar values of the matrix elements of the transition moments in the numerator of this expression, the smaller the energy difference, the larger the dispersion component. Thus, for the D-A-D quadrupolar chromophores, where the excited state involved possesses the lower energy than for the conventional aromatic dimers, the denominator of the dispersion component becomes smaller and as a consequence the total dispersion energy becomes larger for quadrupolar than for non-polar aromatic dimers. (PBE0/6-31+G(d,p) qualitative estimation gives 2.42 eV for squaraine monomer, while for anthracene, phenazine, antraquinone, terphenyl, stilbene and azobenzene the corresponding values are equal respectively 3.30 eV, 3.28 eV, 3.96 eV, 4.40 eV, 3.92 eV and 3.76 eV). On the other hand, as can be expected, the D-A-D systems based on benzooxadiazole or benzothiadiazole moieties exhibit the best similarity to the squaraine stacking dimers considered in the present study. According to our knowledge this is the first indication of enhanced dispersion interaction in stacked quadrupolar molecules due to donor-acceptor charge transfer.

**Figure 10:**
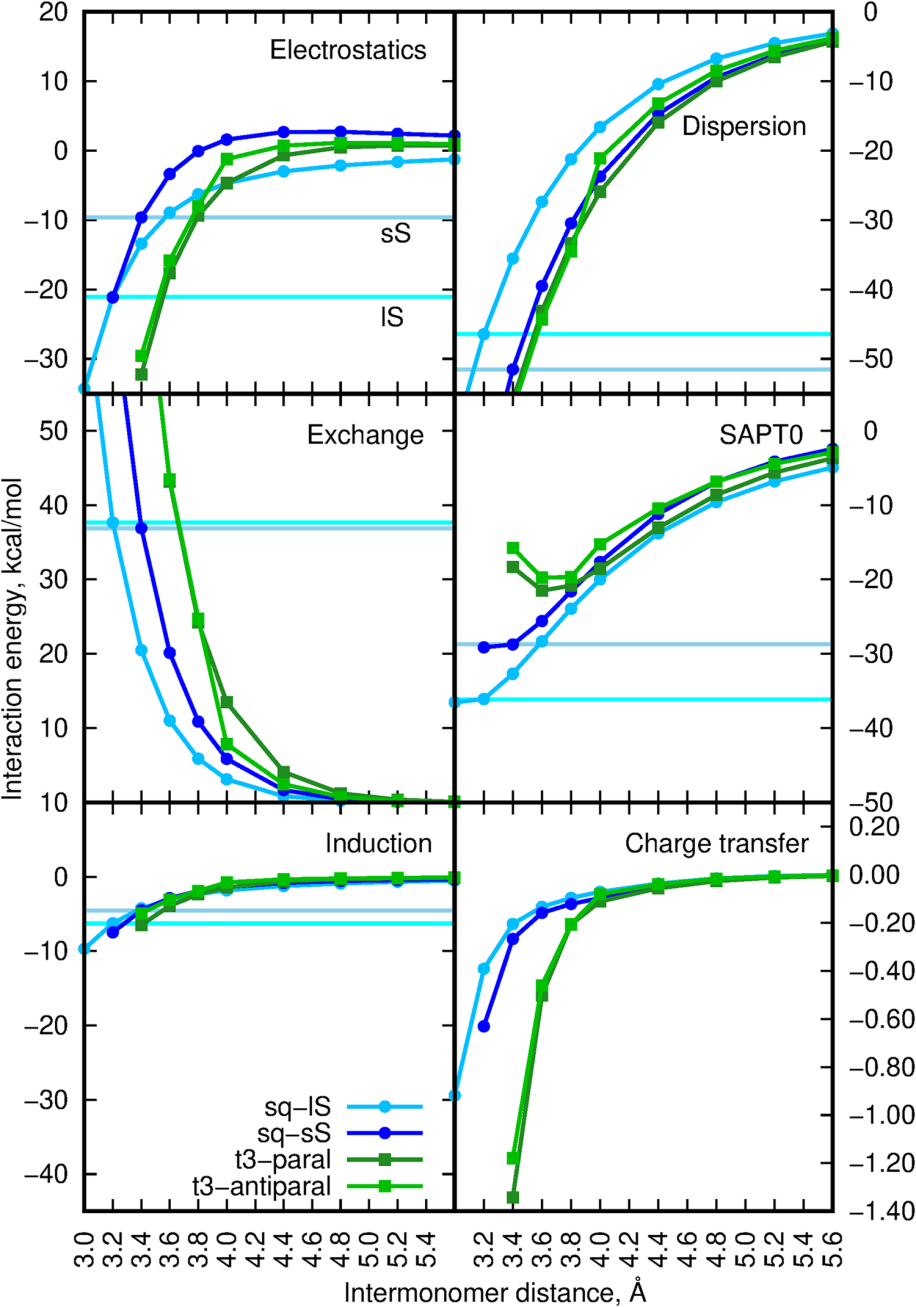
SAPT0 interaction energy and its components for sS and lS squaraine dimers and terthiophene parallel and antiparallel dimers for comparison. The horizontal cyan line depicts the energy value for the lS squaraine dimer, the horizontal blue line refers to the sS squaraine dimer energy values. The light-green polyline illustrate the SCS components. Corresponding numerical values provided in Suppporting Information. For clarity, all components except the charge transfer contribution are presented with the same range of y values.

**Figure 11:**
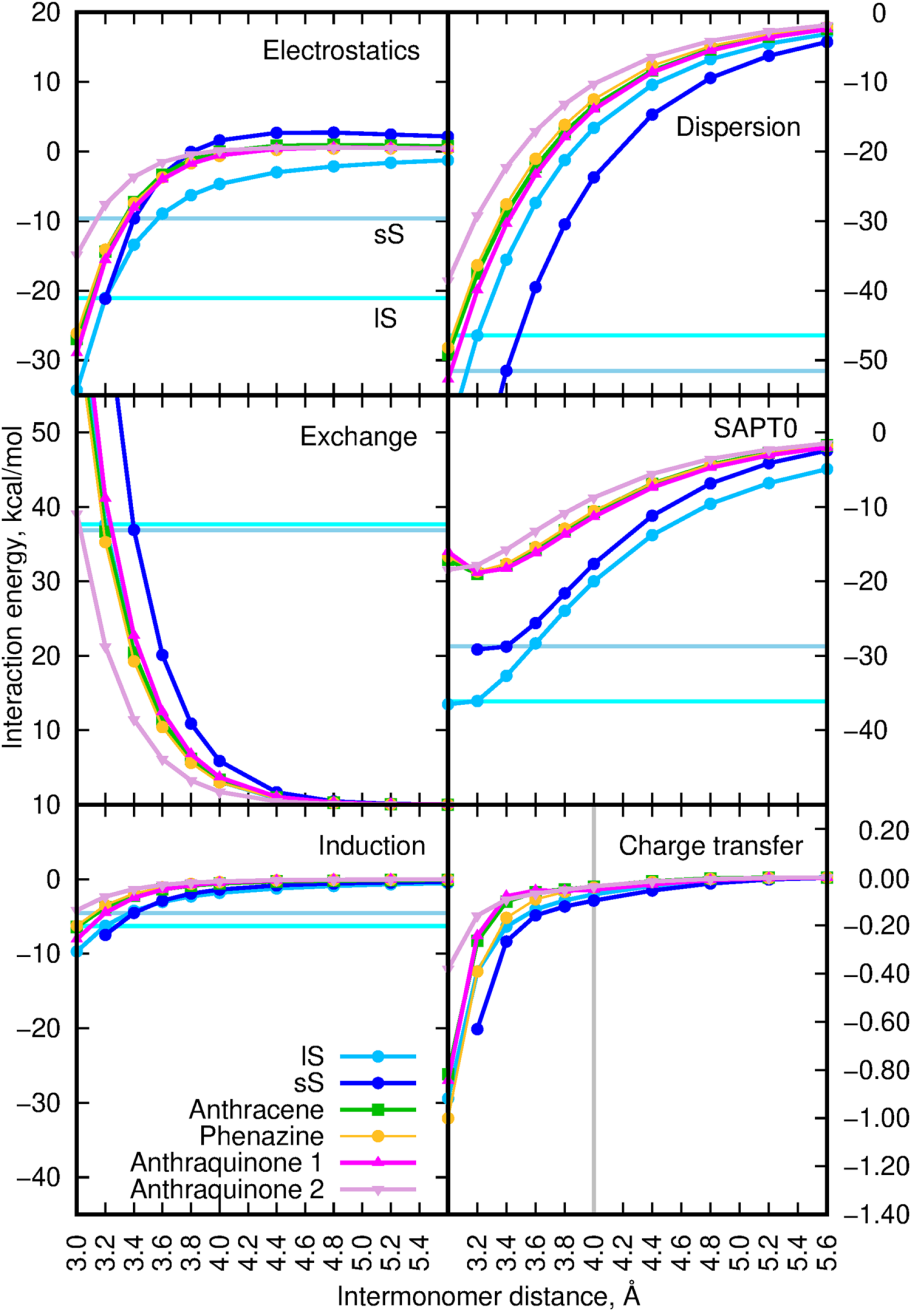
SAPT0 interaction energy and its components for selected cumulated and conjugated dimers with respect to the sS and lS squaraine dimers. The horizontal cyan line depicts the energy value for the lS squaraine dimer, the horizontal blue line refers to the sS squaraine dimer energy values. Corresponding numerical values provided in Suppporting Information. For clarity, all components except the charge transfer are presented with the same range of y values.

The aggregation of $\pi$-conjugated organic molecules is often discussed in the context of their H- or J-aggregation and the following modification of photophysical properties. Such an approach is directly derived from the fundamental model of aggregation proposed by Kasha et al.^39–42^ in 1950s. In the case of molecular aggregates, the electronic excitations become coupled over the whole assembly, thus giving rise to the exciton band splitting, proportional to the intensity of the original monomer transition, and thus a modification of the electronic absorption spectrum of the aggregate with respect to the isolated monomer. The two limiting regimes are distinguished: strong and weak excitonic coupling. The strong coupling regime requires the separability of the electronic and vibrational wave functions for a molecule and manifest itself in the splitting of the absorption band and modification of its vibronic structure. The practical realization of this approach is achieved by replacing the Coulomb potential by multipole expansion usually truncated at the dipole–dipole term. The dependence of this interaction on the inverse third power of the intermolecular distance causes that most of the effects arise from the first several neighbours of the given molecule. On the other in the case of weak coupling, no spectral band structure changes are observed and only band intensity is enhanced or decreased. However, one should remember that one of the basic assumptions of Kasha’s model was that it is limited to the weak interactions between chromophores “through space” in order to ensure the Coulomb coupling. Such a negligible overlap is well realized for the distance between the interacting units larger than 4 Å^43^. Yet, for numerous organic dyes as well as for the squaraine dimers analyzed in the present study, the intermolecular distance often does not exceed 3.5 Å. Therefore one can expect that the spatial overlap between HOMO and LUMO orbitals of the neighbouring molecules is not negligible, thus leading to the significant modification of the photophysics of the stacked $\pi$-electron molecules by the charge transfer interactions. For that reason, for the sS and lS squaraine dimers, additionally the scan over the distance between the two monomers has been performed and the obtained SAPT0 interaction energy components are presented in Fig. 10. In order to compare analyzed squaraine dimers with other aggregating species, anthracene, phenazine, anthraquinone and terthiophene dimers has been also scanned for the 3.0 to 5.6 Å  intermolecular distances (see Fig. 11). Terthiophene was chosen as the small model system of the oligothiophenes, that are known to form H-aggregates. The total SAPT0 interaction energy scan indicates that the distance between the subsystems corresponding to the minimum on the potential energy surface in shifted to the smaller values for squaraines (about 3.2 Å), while it remains in the range of 3.6–3.8 Å  for terthiophene. The larger mutual shift of the monomers in the squaraine dimers results also in the smaller (as for the absolute value) and faster decaying dispersion energy component. These tendencies are similar as for the antraquinone and phenazine dimers. The smaller mutual shift of the squaraine monomers in the stacked dimers on the other hand closely resembles the behaviour of the terthiophene dimers.

For the intermolecular separations corresponding to the minima on the potential energy surface, the charge transfer contribution remain small and quickly decreases with the increase of the intermonomer distance to reach zero for about 5–6 Å, depending on the system. For the indicated distance 4.0 Å, the charge transfer contribution remains on the level of 0.5% of the total SAPT0 interaction energy for all the dimers investigated here.

## Summary

Dimerization of 2,4-bis[4-(dimethyloamine)fenyl]cyclobutane-1,3-diole molecules is investigated with quantum chemistry approaches. Four different mutual orientations are analyzed: two stacked ones and two with hydrogen-bond-like contacts. The stacked dimers are governed by dispersion interactions and remain the most stable. The perpendicular and approximately coplanar systems on the other hand possess mixed nature with a electrostatic interaction energy component of similar quantity to the dispersion component and remain significantly less stable than stacked dimers.

Because the DMASQ molecules exhibit interesting interaction-induced properties such as non-zero second harmonic generation in the aggregates, it is crucial to adequately describe their mutual interaction even in the sizeable complexes. Therefore the wide range of density functionals have been included into considerations and the reference interaction energy estimates have been obtained within the MP2C and (SCS-)SAPT0 approaches. The particularly good behaviour for supermolecular interaction energy and almost negligible basis set dependence can be noticed for B3LYP-D3, B3LYP-D3BJ, B97-D3BJ and M05-2X-D3 functionals applied with counterpoise correction. The traditional MP2 is confirmed to significantly overestimate the dispersion-governed dimers and give a dramatic dependence of the generated results on the basis set quality, therefore it should be avoided in the case of squaraine dimers.

The most appealing results of the present study confirm the high dispersion energy component for the analyzed quadrupolar dimers in comparison to the conventionally investigated aromatic dimers such as anthracene or phenazine. The value of the monomer displacement influences significantly the dispersion-to-electrostatic energy component ratio, thus causing that the dispersion component in the large displacement stacked squaraine dimers resemble to the high extend this contribution for antraquinone or phenazine dimers. On the other hand the modification of the dispersion energy component with the increasing intermonomer distance for the smaller displacement dimers reproduce the tendencies observed for the thethiophene H-aggregates.

The nature of the intermolecular interactions in the aggregates of the squaraines is crucial from the point of view of their photophysical properties. These have been previously studied by the current authors for two squaraine systems, namely DMASQ and 2,4-bis(aminophenyl) squaraine (DASQ)^44^. They have been chosen as the exemplary molecules representing different hydrogen-bond donating/accepting ability: DASQ in contrast to DMASQ possesses the strong hydrogen bond donor. The lack of such a central amino group in DMASQ determines the nature and stability of the obtained aggregates and significantly modifies the absorption spectrum changes upon aggregation. For DASQ simple batochromic shift and increase of the intensity is noticed upon aggregation, while for DMASQ analyzed in the present study, additionally strong changes of the spectral shape and additional bands are observed for staircase stacked aggregates^44^.

The aggregation phenomena of squaraines investigated in the exciton model or within the essential states model provide the numerous aberrance from the widely applied aggregation classification, exhibiting the existence of the ”non-fluorescent J-aggregates”^45,46^ or aggregation-enhanced two-photon response. The conclusion that the simple H- and J-classification is hardly applicable for the highly polarizable molecules indicates that there is still a need for further study of squaraine aggregation.

## Supplementary information

Supplementary Information.
