# Supplementary material for: Nature of intermolecular interaction in squaraine dimers
Source: Sci Rep. 2020 Nov 12;10:19670. doi: 10.1038/s41598-020-76631-z (PMC7665223; doi:10.1038/s41598-020-76631-z)
Supplement: Supplementary file 1 — Supplementary Information. [file 41598_2020_76631_MOESM1_ESM.pdf]

Supporting Information: Nature of intermolecular interaction in squaraine dimers

Anna Kaczmarek-Kędziera,*,† Piotr Żuchowski,‡ and Dariusz Kędziera†

†*Faculty of Chemistry, Nicolaus Copernicus University in Torun, Gagarina 7, 87–100 Toruń, Poland*

‡*Institute of Physics, Nicolaus Copernicus University in Torun, Grudziądzka 5, 87–100 Toruń, Poland*

E-mail: teoadk@chem.umk.pl

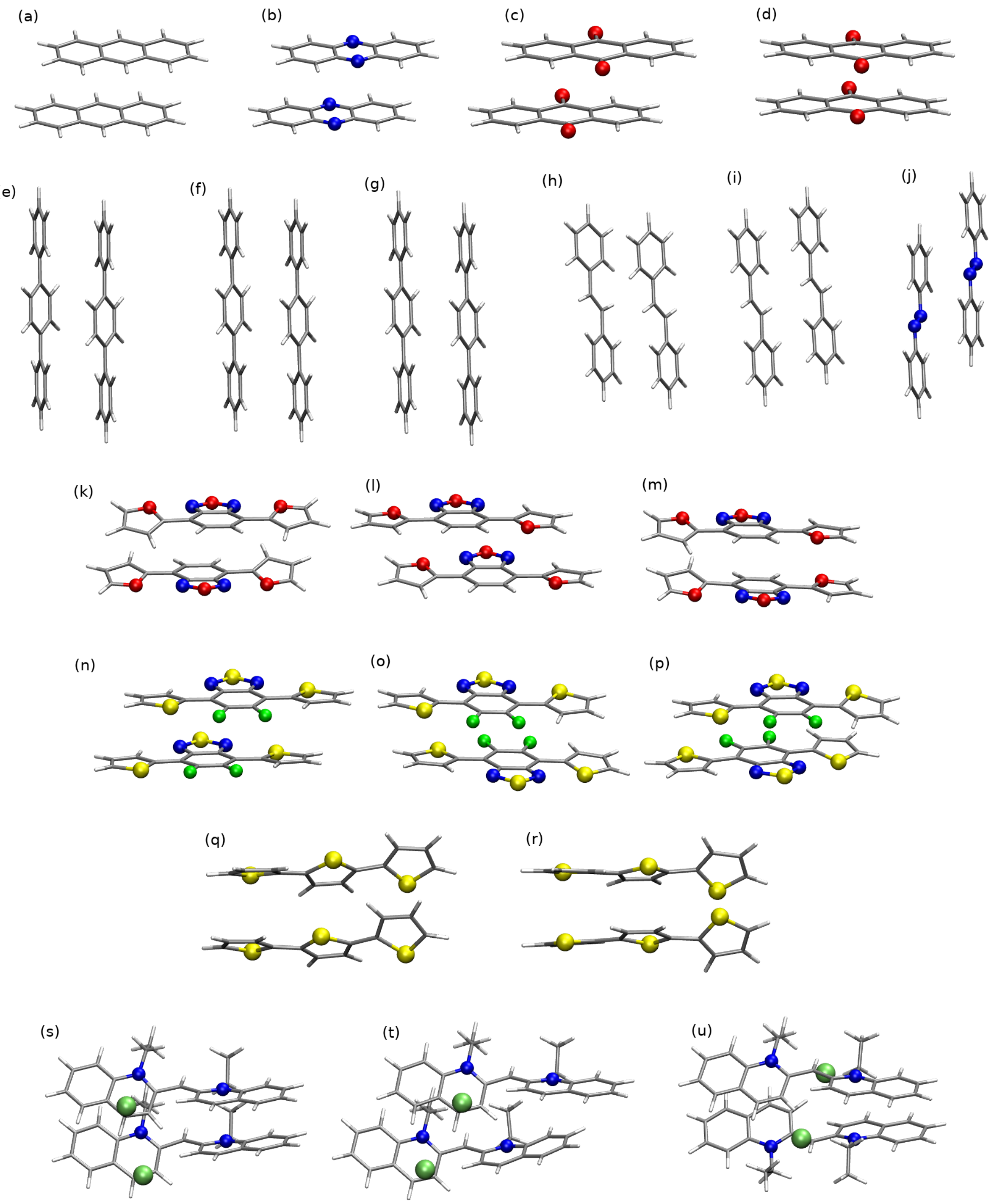

Figure S1: B3LYP-D3/cc-pVDZ optimized dimers of: (a) antracene, (b) phenazine, (c) and (d) anthraquinone, (e)–(g) terphenyl, (h) and (i) stilbene, (j) azobenzene, (k)–(m) benzooxadiazole, (n)–(p) fluorinated benzothiadiazole, (q) and (r) terthiophene and (s)–(u) pseudoisocyanine chloride. For easier comparison, heteroatoms are depicted with CPK representation and corresponding color coding: nirogen – blue, oxygen – red, sulphur – yellow, fluorine – green

Table S1: SAPT0/aug-cc-pVDZ interaction energies and its components for the lS and sS squaraine and parallel and antiparallel terthiophene (T3, (q) and (r) structures in Fig. S1, respectively) optimized dimer structures (i. e. minima) [kcal/mol]

Dimer	lS	sS	T3-parall (q)	T3-antiparall (r)
Electrostatics	-21.04	-9.62	-9.36	-8.00
$E^{(10)}_{elst,r}$	-21.04	-9.62	-9.36	-8.00
Exchange	37.67	36.90	24.24	24.71
$E^{(10)}_{exch}$	37.67	36.90	24.24	24.71
$E^{(10)}_{exch}(S^2)$	37.66	36.88	24.22	24.71
Induction	-6.26	-4.51	-2.36	-1.88
$E^{(20)}_{ind,r}$	-16.21	-15.29	-11.93	-10.59
$E^{(20)}_{exch-ind,r}$	13.50	13.51	11.20	9.99
$\delta^{HF}{}_r(2)$	-3.55	-2.73	-1.63	-1.29
Dispersion	-46.45	-51.51	-33.34	-34.49
$E^{(20)}_{disp}$	-52.30	-57.63	-37.79	-39.00
$E^{(20)}_{exch-disp}$	5.85	6.12	4.45	4.51
SCS Dispersion	-36.23	-40.17	-26.01	-26.92
$E^{(20)}_{disp}(SS)$	-26.15	-28.81	-18.89	-19.50
$E^{(20)}_{disp}(OS)$	-26.15	-28.81	-18.89	-19.50
$E^{(20)}_{exch-disp}(SS)$	3.64	3.84	2.74	2.81
$E^{(20)}_{exch-disp}(OS)$	2.21	2.28	1.71	1.70
Total HF	10.37	22.77	12.51	14.82
Total SAPT0	-36.08	-28.74	-20.83	-19.67
Total SCS-SAPT0	-25.86	-17.40	-13.50	-12.10
Charge transfer	-0.39	-0.27	-0.21	-0.21
% CT in Total SAPT0	1.08	0.94	1.01	1.07
Disp/Elst ratio	2.21	5.35	3.56	4.31

Table S2: SAPT0/aug-cc-pVDZ interaction energies and its components for anthracene, phenazine and anthraquinone optimized dimer structures (i. e. minima corresponding to (a)–(d) panels from Fig. S1) [kcal/mol]

Dimer	Anthracene(a)	Phenazine(b)	Anthraquinone(c)	Anthraquinone(d)
Electrostatics	-7.31	-7.41	-8.11	-7.58
$E^{(10)}_{elst,r}$	-7.31	-7.41	-8.11	-7.58
Exchange	20.42	19.28	22.76	21.28
$E^{(10)}_{exch}$	20.42	19.28	22.76	21.28
$E^{(10)}_{exch}(S^2)$	20.40	19.25	22.71	21.24
Induction	-2.23	-1.92	-2.48	-2.32
$E^{(20)}_{ind,r}$	-8.81	-8.13	-10.33	-9.15
$E^{(20)}_{exch-ind,r}$	8.21	7.70	9.60	8.36
$\delta^{HF}{}_r(2)$	-1.63	-1.49	-1.76	-1.52
Dispersion	-28.80	-27.58	-30.41	-29.20
$E^{(20)}_{disp}$	-32.77	-31.07	-34.29	-32.80
$E^{(20)}_{exch-disp}$	3.97	3.48	3.88	3.60
SCS Dispersion	-22.50	-21.52	-23.74	-22.77
$E^{(20)}_{disp}(SS)$	-16.38	-15.53	-17.14	-16.40
$E^{(20)}_{disp}(OS)$	-16.38	-15.53	-17.14	-16.40
$E^{(20)}_{exch-disp}(SS)$	2.47	2.17	2.42	2.25
$E^{(20)}_{exch-disp}(OS)$	1.50	1.32	1.45	1.36
Total HF	10.89	9.94	12.16	11.38
Total SAPT0	-17.91	-17.64	-18.25	-17.82
Total SCS-SAPT0	-11.61	-11.57	-11.58	-11.39
Charge transfer	-0.10	-0.17	-0.08	-0.16
% CT in Total SAPT0	0.56	0.96	0.44	0.90
Disp/Elst ratio	3.94	3.72	3.75	3.85

Table S3: SAPT0/aug-cc-pVDZ interaction energies and its components for terphenyl, stilbene and azobenzene optimized dimer structures (i. e. minima corresponding to (e)–(j) panels from Fig. S1) [kcal/mol]

	Terphenyl			Stilbene		Azobenzene
Dimer	(e)	(f)	(g)	(h)	(i)	(j)
Electrostatics	-6.28	-6.44	-6.11	-6.85	-6.69	-7.08
$E^{(10)}_{elst,r}$	-6.28	-6.44	-6.11	-6.85	-6.69	-7.08
Exchange	22.21	22.42	21.65	19.13	18.70	19.01
$E^{(10)}_{exch}$	22.21	22.42	21.65	19.13	18.70	19.01
$E^{(10)}_{exch}(S^2)$	22.21	22.40	21.64	19.10	18.66	18.96
Induction	-2.08	-2.11	-2.05	-2.20	-2.05	-2.02
$E^{(20)}_{ind,r}$	-9.13	-9.29	-8.89	-8.42	-8.24	-8.43
$E^{(20)}_{exch-ind,r}$	8.48	8.63	8.24	7.74	7.56	7.99
$\delta^{HF}{}_{r}(2)$	-1.43	-1.45	-1.41	-1.51	-1.37	-1.59
Dispersion	-32.95	-32.94	-32.49	-24.99	-24.71	-24.70
$E^{(20)}_{disp}$	-37.29	-37.32	-36.73	-28.58	-28.25	-28.06
$E^{(20)}_{exch-disp}$	4.34	4.37	4.24	3.59	3.53	3.36
SCS Dispersion	-25.74	-25.74	-25.37	-19.55	-19.33	-19.29
$E^{(20)}_{disp}(SS)$	-18.64	-18.66	-18.36	-14.29	-14.12	-14.03
$E^{(20)}_{disp}(OS)$	-18.64	-18.66	-18.36	-14.29	-14.12	-14.03
$E^{(20)}_{exch-disp}(SS)$	2.72	2.74	2.66	2.24	2.21	2.08
$E^{(20)}_{exch-disp}(OS)$	1.62	1.63	1.58	1.34	1.32	1.28
Total HF	13.86	13.87	13.49	10.08	9.96	9.91
Total SAPT0	-19.09	-19.07	-19.00	-14.90	-14.76	-14.80
Total SCS-SAPT0	-11.88	-11.87	-11.88	-9.46	-9.38	-9.38
Charge transfer	-0.20	-0.20	-0.20	-0.18	-0.16	-0.21
% CT in Total SAPT0	1.05	1.05	1.05	1.21	1.08	1.42
Disp/Elst ratio	5.25	5.11	5.32	3.65	3.69	3.49

Table S4: SAPT0/aug-cc-pVDZ interaction energies and its components for benzoxadiazole and benzothiadiazole optimized dimer structures (i. e. minima corresponding to (k)–(p) panels from Fig. S1) [kcal/mol]

	Benzooxadiazole			Benzothiadiazole		
Dimer	(k)	(l)	(m)	(n)	(o)	(p)
Electrostatics	-15.51	-8.00	-13.18	-12.44	-13.72	-12.66
$E^{(10)}_{elst,r}$	-15.51	-8.00	-13.18	-12.44	-13.72	-12.66
Exchange	29.78	23.88	28.26	32.14	35.00	33.93
$E^{(10)}_{exch}$	29.78	23.88	28.26	32.14	35.00	33.93
$E^{(10)}_{exch}(S^2)$	29.75	23.86	28.23	32.13	34.98	33.93
Induction	-3.42	-1.94	-2.78	-2.75	-3.54	-3.30
$E^{(20)}_{ind,r}$	-14.44	-11.74	-13.65	-15.89	-16.61	-16.44
$E^{(20)}_{exch-ind,r}$	13.24	11.10	12.81	14.98	15.54	15.44
$\delta^{HF}{}_r(2)$	-2.22	-1.30	-1.94	-1.84	-2.47	-2.30
Dispersion	-39.14	-34.78	-37.83	-46.45	-48.89	-48.67
$E^{(20)}_{disp}$	-44.23	-38.97	-42.70	-51.98	-54.84	-54.52
$E^{(20)}_{exch-disp}$	5.08	4.19	4.88	5.53	5.95	5.85
SCS Dispersion	-30.53	-27.10	-29.51	-36.18	-38.08	-37.90
$E^{(20)}_{disp}(SS)$	-22.11	-19.48	-21.35	-25.99	-27.42	-27.26
$E^{(20)}_{disp}(OS)$	-22.11	-19.48	-21.35	-25.99	-27.42	-27.26
$E^{(20)}_{exch-disp}(SS)$	3.14	2.60	3.02	3.42	3.67	3.60
$E^{(20)}_{exch-disp}(OS)$	1.94	1.59	1.85	2.10	2.28	2.26
Total HF	10.85	13.93	12.29	16.95	17.74	17.97
Total SAPT0	-28.29	-20.85	-25.53	-29.50	-31.15	-30.70
Total SCS-SAPT0	-19.68	-13.17	-17.21	-19.23	-20.34	-19.92
Charge transfer	-0.22	-0.20	-0.17	-0.20	-0.21	-0.29
% CT in Total SAPT0	0.78	0.96	0.66	0.68	0.67	0.94
Disp/Elst ratio	2.52	4.34	2.87	3.73	3.56	3.84

Table S5: SAPT0/aug-cc-pVDZ interaction energies and its components for pseudocyanine chloride optimized dimer structures (i. e. minima corresponding to (s)–(u) panels from Fig. S1) [kcal/mol]

	Pseudocyanine chloride		
Dimer	(s)	(t)	(u)
Electrostatics	-15.22	-16.28	-54.76
$E^{(10)}_{elst,r}$	-15.22	-16.28	-54.76
Exchange	37.94	35.20	71.42
$E^{(10)}_{exch}$	37.94	35.20	71.42
$E^{(10)}_{exch}(S^2)$	37.88	35.12	71.03
Induction	-7.05	-7.60	-22.60
$E^{(20)}_{ind,r}$	-18.70	-17.48	-41.19
$E^{(20)}_{exch-ind,r}$	14.86	13.24	27.76
$\delta^{HF}{}_r(2)$	-3.21	-3.36	-9.17
Dispersion	-49.11	-43.04	-63.25
$E^{(20)}_{disp}$	-55.31	-48.40	-72.83
$E^{(20)}_{exch-disp}$	6.20	5.36	9.57
SCS Dispersion	-38.31	-33.57	-49.51
$E^{(20)}_{disp}(SS)$	-27.65	-24.20	-36.41
$E^{(20)}_{disp}(OS)$	-27.65	-24.20	-36.41
$E^{(20)}_{exch-disp}(SS)$	3.87	3.34	5.96
$E^{(20)}_{exch-disp}(OS)$	2.33	2.02	3.61
Total HF	15.67	11.31	-5.94
Total SAPT0	-33.44	-31.72	-69.19
Total SCS-SAPT0	-22.65	-22.26	-55.45
Charge transfer	-0.68	-0.82	-2.77
% CT in Total SAPT0	2.03	2.58	4.00
Disp/Elst ratio	3.23	2.64	1.16

Table S6: Scan of the SAPT0/aug-cc-pVDZ interaction energy and its components for the sS squaraine dimer structures [kcal/mol]

Dimer	3.0	3.2	3.4	3.6	3.8	4.0	4.4	4.8	5.2	5.6
Electrostatics	-42.08	-21.16	-9.62	-3.38	-0.10	1.58	2.68	2.70	2.44	2.16
$E^{(10)}_{elst,r}$	-42.08	-21.16	-9.62	-3.38	-0.10	1.58	2.68	2.70	2.44	2.16
Exchange	120.99	67.13	36.90	20.13	10.91	5.88	1.68	0.47	0.13	0.03
$E^{(10)}_{exch}$	120.99	67.13	36.90	20.13	10.91	5.88	1.68	0.47	0.13	0.03
$E^{(10)}_{exch}(S^2)$	120.33	66.97	36.88	20.13	10.91	5.88	1.68	0.47	0.13	0.03
Induction	-12.82	-7.46	-4.51	-2.86	-1.92	-1.37	-0.82	-0.57	-0.43	-0.33
$E^{(20)}_{ind,r}$	-53.68	-28.57	-15.29	-8.29	-4.63	-2.73	-1.19	-0.70	-0.49	-0.36
$E^{(20)}_{exch-ind,r}$	49.99	26.18	13.51	6.87	3.46	1.74	0.46	0.14	0.05	0.02
$\delta^{HF}{}_r(2)$	-9.13	-5.06	-2.73	-1.44	-0.75	-0.38	-0.09	-0.01	0.01	0.01
Dispersion	-89.42	-67.65	-51.51	-39.49	-30.49	-23.72	-14.72	-9.44	-6.26	-4.27
$E^{(20)}_{disp}$	-105.81	-77.73	-57.63	-43.15	-32.66	-24.99	-15.14	-9.58	-6.30	-4.29
$E^{(20)}_{exch-disp}$	16.39	10.09	6.12	3.66	2.17	1.27	0.42	0.14	0.04	0.01
SCS Dispersion	-70.22	-52.93	-40.17	-30.70	-23.64	-18.35	-11.34	-7.26	-4.81	-3.28
$E^{(20)}_{disp}(SS)$	-52.90	-38.87	-28.81	-21.58	-16.33	-12.50	-7.57	-4.79	-3.15	-2.14
$E^{(20)}_{disp}(OS)$	-52.90	-38.87	-28.81	-21.58	-16.33	-12.50	-7.57	-4.79	-3.15	-2.14
$E^{(20)}_{exch-disp}(SS)$	10.12	6.28	3.84	2.32	1.38	0.88	0.28	0.09	0.03	0.01
$E^{(20)}_{exch-disp}(OS)$	6.27	3.81	2.28	1.34	0.78	0.45	0.14	0.04	0.01	0.00
Total HF	66.09	38.52	22.77	13.89	8.90	6.09	3.54	2.59	2.14	1.86
Total SAPT0	-23.33	-29.13	-28.74	-25.60	-21.59	-17.64	-11.18	-6.85	-4.12	-2.42
Total SCS-SAPT0	-4.13	-14.42	-17.40	-16.81	-14.74	-12.26	-7.80	-4.67	-2.67	-1.42
Charge transfer	-1.70	-0.63	-0.27	-0.16	-0.12	-0.10	-0.06	-0.03	-0.01	0.00
% CT in Total SAPT0	7.29	2.17	0.93	0.62	0.56	0.55	0.50	0.39	0.25	0.12
Disp/Elst ratio	2.13	3.20	5.35	11.68	350.12	-15.00	-5.48	-3.50	-2.56	-1.98

Table S7: Scan of the SAPT0/aug-cc-pVDZ interaction energy and its components for the lS squaraine dimer structures [kcal/mol]

Dimer	3.0	3.2	3.4	3.6	3.8	4.0	4.4	4.8	5.2	5.6
Electrostatics	-34.30	-21.04	-13.39	-8.93	-6.30	-4.69	-2.98	-2.14	-1.64	-1.29
$E^{(10)}_{elst,r}$	-34.30	-21.04	-13.39	-8.93	-6.30	-4.69	-2.98	-2.14	-1.64	-1.29
Exchange	68.58	37.67	20.48	11.04	5.90	3.14	0.87	0.24	0.06	0.02
$E^{(10)}_{exch}$	68.58	37.67	20.48	11.04	5.90	3.14	0.87	0.24	0.06	0.02
$E^{(10)}_{exch}(S^2)$	68.46	37.66	20.49	11.04	5.91	3.14	0.87	0.24	0.06	0.02
Induction	-9.69	-6.26	-4.24	-3.04	-2.30	-1.81	-1.23	-0.90	-0.68	-0.52
$E^{(20)}_{ind,r}$	-29.77	-16.21	-9.05	-5.27	-3.27	-2.20	-1.25	-0.85	-0.63	-0.48
$E^{(20)}_{exch-ind,r}$	26.21	13.50	6.86	3.45	1.72	0.87	0.24	0.08	0.03	0.01
$\delta^{HF}{}_r(2)$	-6.13	-3.55	-2.06	-1.22	-0.75	-0.48	-0.23	-0.13	-0.08	-0.05
Dispersion	-61.09	-46.45	-35.54	-27.38	-21.24	-16.62	-10.42	-6.76	-4.53	-3.13
$E^{(20)}_{disp}$	-70.76	-52.30	-39.03	-29.43	-22.44	-17.31	-10.65	-6.84	-4.56	-3.14
$E^{(20)}_{exch-disp}$	9.67	5.85	3.49	2.06	1.20	0.69	0.22	0.07	0.02	0.01
SCS Dispersion	-47.81	-36.23	-27.63	-21.23	-16.44	-12.83	-8.02	-5.20	-3.48	-2.40
$E^{(20)}_{disp}(SS)$	-35.38	-26.15	-19.51	-14.72	-11.22	-8.65	-5.32	-3.42	-2.28	-1.57
$E^{(20)}_{disp}(OS)$	-35.38	-26.15	-19.51	-14.72	-11.22	-8.65	-5.32	-3.42	-2.28	-1.57
$E^{(20)}_{exch-disp}(SS)$	5.96	3.64	2.19	1.31	0.77	0.45	0.15	0.05	0.02	0.00
$E^{(20)}_{exch-disp}(OS)$	3.71	2.21	1.30	0.75	0.43	0.24	0.07	0.02	0.01	0.00
Total HF	24.59	10.37	2.85	-0.94	-2.69	-3.36	-3.34	-2.80	-2.25	-1.79
Total SAPT0	-36.49	-36.08	-32.68	-28.31	-23.93	-19.98	-13.77	-9.57	-6.78	-4.92
Total SCS-SAPT0	-23.22	-25.86	-24.78	-22.16	-19.12	-16.19	-11.37	-8.00	-5.73	-4.19
Charge transfer	-0.92	-0.39	-0.20	-0.13	-0.10	-0.07	-0.04	-0.02	0.00	0.00
% CT in Total SAPT0	2.52	1.09	0.63	0.47	0.40	0.36	0.27	0.17	0.00	0.00
Disp/Elst ratio	1.78	2.21	2.65	3.06	3.37	3.55	3.50	3.16	2.77	2.43

Table S8: PSI4 supermolecular counterpoise-corrected interaction energies for the dimer structures optimized within the B3LYP-GD3/cc-pVDZ approach, [kcal/mol]

Dimer	lS	sS	lhb	lhb-B3LYP
		cc-pVDZ #bf 872		
B3LYP	4.93	17.21	-3.14	-6.92
B3LYP-D2	-26.71	-17.42	-14.91	-13.11
B3LYP-D3	-24.24	-15.43	-15.01	-13.62
B3LYP-D3BJ	-25.55	-17.64	-14.46	-12.95
B3LYP-CHG	-18.15	-9.87	-12.11	-11.66
B97-D2	-26.04	-17.61	-13.76	-11.72
B97-D3	-23.69	-14.74	-14.00	-12.30
B97-D3BJ	-26.36	-18.90	-14.04	-11.96
B2PLYP	-6.53	4.12	-6.85	-8.94
B2PLYP-D2	-23.10	-14.01	-13.01	-12.19
B2PLYP-D3	-21.68	-12.62	-13.18	-12.56
B2PLYP-D3BJ	-22.21	-14.00	-12.75	-12.12
DSD-BLYP	-22.81	-14.06	-12.56	-11.95
DSD-PBEPBE	-23.21	-14.31	-12.43	-11.64
DSD-PBEP86	-22.20	-13.32	-12.38	-11.81
wB97X-D	-26.78	-19.67	-14.08	-12.03
DLDF	19.93	29.84	3.57	-2.72
DLDF+D	-22.12	-15.22	-11.55	-10.65
DLDF+D09	-23.38	-18.19	-10.94	-10.38
M05	-6.98	3.10	-7.90	-9.79
M05-2X	-18.45	-7.60	-10.76	-10.96
M05-2X-D3	-24.39	-15.02	-14.18	-13.21
M05-D3	-20.53	-12.55	-14.11	-13.52
MP2	-23.31	-16.00	-11.32	-10.92
MP2C	-14.08	-5.16	-9.59	-10.46
HF	9.99	22.05	-0.34	-5.53
SAPT0	-25.74	-17.58	-13.49	-13.02
SCS-SAPT0	-17.67	-8.63	-10.49	-11.29

Table S9: PSI4 supermolecular counterpoise-corrected interaction energies for the dimer structures optimized within the B3LYP-GD3/cc-pVDZ approach, [kcal/mol]

Dimer	lS	sS	lhb	lhb-B3LYP
		cc-pVTZ #bf 2000		
B3LYP	4.51	16.92	-3.51	-7.21
B3LYP-D2	-27.14	-17.71	-15.28	-13.40
B3LYP-D3	-24.66	-15.73	-15.38	-13.90
B3LYP-D3BJ	-25.97	-17.94	-14.83	-13.24
B3LYP-CHG	-18.57	-10.16	-12.48	-11.95
B97-D2	-26.11	-17.53	-14.05	-11.96
B97-D3	-23.76	-14.66	-14.28	-12.55
B97-D3BJ	-26.42	-18.82	-14.32	-12.21
B2PLYP	-9.93	0.67	-8.41	-9.83
B2PLYP-D2	-26.50	-17.47	-14.57	-13.07
B2PLYP-D3	-25.08	-16.07	-14.74	-13.44
B2PLYP-D3BJ	-25.61	-17.46	-14.31	-13.00
DSD-BLYP	-27.66	-19.00	-14.72	-13.14
DSD-PBEPBE	-26.49	-17.48	-14.09	-12.58
DSD-PBEP86	-26.34	-17.49	-14.34	-12.91
wB97X-D	-26.98	-19.43	-14.55	-12.37
DLDF	19.00	28.77	3.16	-3.13
DLDF+D	-23.05	-16.29	-11.96	-11.06
DLDF+D09	-24.30	-19.26	-11.35	-10.80
M05	-6.25	3.92	-7.92	-9.88
M05-2X	-18.75	-7.40	-11.36	-11.44
M05-2X-D3	-24.70	-14.83	-14.78	-13.69
M05-D3	-19.80	-11.73	-14.13	-13.60
MP2	-32.41	-25.35	-15.27	-13.08
HF	10.41	22.70	-0.27	-5.40
SAPT0	-34.33	-26.83	-17.06	-14.78
SCS-SAPT0	-24.44	-15.87	-13.35	-12.68

Table S10: PSI4 supermolecular counterpoise-corrected interaction energies for the dimer structures optimized within the B3LYP-GD3/cc-pVDZ approach, [kcal/mol]. Pochyłe energie uzyskane w plikach o nazwie 2.k.hb - w zasadzie ta sama struktura

Dimer	lS	sS	lhb	lhb-B3LYP
		jun-cc-pVDZ #bf 1064		
B3LYP	4.74	17.32	-3.22	-9.53
B3LYP-D2	-26.90	-17.30	-14.99	-12.77
B3LYP-D3	-24.43	-15.32	-15.09	-13.15
B3LYP-D3BJ	-25.74	-17.53	-14.54	-12.70
B3LYP-CHG	-18.34	-9.76	-12.19	-11.98
B97-D2	-26.18	-17.40	-13.87	-12.01
B97-D3	-23.82	-14.52	-14.10	-12.59
B97-D3BJ	-26.49	-18.69	-14.14	-12.25
B2PLYP	-8.17	2.51	-7.46	-9.53
B2PLYP-D2	-24.74	-15.63	-13.63	-12.77
B2PLYP-D3	-23.32	-14.23	-13.79	-13.15
B2PLYP-D3BJ	-23.85	-15.62	-13.36	-12.70
DSD-BLYP	-25.10	-16.44	-13.42	-12.66
DSD-PBEPBE	-24.91	-15.97	-13.11	-12.26
DSD-PBEP86	-24.29	-15.48	-13.20	-12.52
wB97X-D	-27.40	-19.90	-14.39	-12.45
DLDF	19.77	29.95	3.43	-3.13
DLDF+D	-22.27	-15.11	-11.70	-11.06
DLDF+D09	-23.53	-18.08	-11.09	-10.79
M05	-7.08		-8.13	-10.31
M05-2X	-19.27	-8.17	-11.13	-11.49
M05-2X-D3	-25.21	-15.59	-14.55	-13.75
M05-D3	-20.64		-14.34	-14.04
MP2	-27.47	-20.56	-12.84	-11.98
HF	10.32			
SAPT0	-29.68	-22.03	-15.00	-13.95
SCS-SAPT0	-20.74	-12.03	-11.69	-12.08

Table S11: PSI4 supermolecular counterpoise-corrected interaction energies for the dimer structures optimized within the B3LYP-GD3/cc-pVDZ approach, [kcal/mol]. Pochyłe energie uzyskane w plikach o nazwie 2.k.hb - w zasadzie ta sama struktura

Dimer	lS	sS	lhb	lhb-B3LYP
	jun-cc-pVTZ #bf 2432			
B3LYP	4.55	16.91	-3.49	-7.26
B3LYP-D2	-27.09	-17.72	-15.26	-13.45
B3LYP-D3	-24.62	-15.74	-15.36	-13.96
B3LYP-D3BJ	-25.93	-17.95	-14.81	-13.29
B3LYP-CHG	-18.53	-10.18	-12.46	-12.00
B97-D2	-26.13	-17.59	-14.05	-12.03
B97-D3	-23.77	-14.72	-14.28	-12.62
B97-D3BJ	-26.44	-18.89	-14.33	-12.28
B2PLYP	-10.75	-0.24	-8.78	-10.16
B2PLYP-D2	-27.33	-18.38	-14.95	-13.40
B2PLYP-D3	-25.90	-16.99	-15.11	-13.78
B2PLYP-D3BJ	-26.44	-18.38	-14.68	-13.33
DSD-BLYP	-28.92	-20.37	-15.28	-13.61
DSD-PBEPBE	-27.53	-18.62	-14.54	-12.96
DSD-PBEP86	-27.51	-18.77	-14.84	-13.34
wB97X-D	-27.05	-19.63	-14.59	-12.44
DLDF	19.13	28.92	3.19	-3.20
DLDF+D	-22.92	-16.14	-11.94	-11.13
DLDF+D09	-24.18	-19.11	-11.33	-10.87
M05	-6.26	3.68	-7.97	-10.00
M05-2X	-18.79	-7.52	-11.39	-11.53
M05-2X-D3	-24.73	-14.95	-14.81	-13.78
M05-D3	-19.82	-11.98	-14.18	-13.73
MP2	-34.91	-28.02	-16.34	-13.90
HF	10.43	22.68	-0.31	-5.48
SAPT0	-36.86	-29.63	-18.15	-15.56
SCS-SAPT0	-26.50	-18.14	-14.24	-13.33

Table S12: PSI4 supermolecular counterpoise-corrected interaction energies for the dimer structures optimized within the B3LYP-GD3/cc-pVDZ approach, [kcal/mol]. Pochyłe energie uzyskane w plikach o nazwie 2.k.hb - w zasadzie ta sama struktura

Dimer	lS	sS	lhb	lhb-B3LYP
		aug-cc-pVDZ #bf 1464		
B3LYP	4.74	17.29	-3.46	-7.27
B3LYP-D2	-26.90	-17.34	-15.23	-13.46
B3LYP-D3	-24.42	-15.35	-15.33	-13.96
B3LYP-D3BJ	-25.74	-17.56	-14.78	-13.30
B3LYP-CHG	-18.34	-9.79	-12.43	-12.01
B97-D2	-26.20	-17.49	-14.13	-12.05
B97-D3	-23.85	-14.62	-14.36	-12.64
B97-D3BJ	-26.52	-18.79	-14.41	-12.30
B2PLYP	-10.26	0.32	-8.65	-10.12
B2PLYP-D2	-26.84	-17.82	-14.81	-13.36
B2PLYP-D3	-25.42	-16.42	-14.98	-13.73
B2PLYP-D3BJ	-25.95	-17.81	-14.54	-13.29
DSD-BLYP	-28.29	-19.74	-15.08	-13.52
DSD-PBEPBE	-27.36	-18.50	-14.46	-12.90
DSD-PBEP86	-27.10	-18.40	-14.70	-13.27
wB97X-D	-27.72	-20.32	-14.87	-12.52
DLDF	19.72	29.83	3.24	-3.17
DLDF+D	-22.33	-15.23	-11.88	-11.11
DLDF+D09	-23.59	-18.20	-11.27	-10.84
M05	-6.96	3.04	-8.31	-10.26
M05-2X	-19.69	-8.59	-11.66	-11.57
M05-2X-D3	-25.63	-16.02	-15.09	-13.83
M05-D3	-20.52	-12.61	-14.53	-13.99
MP2	-33.76	-27.00	-15.87	-13.65
MP2C	-22.80	-14.22	-13.83	-13.19
HF	10.37			
SAPT0	-36.08	-28.74	-17.96	-15.44
SCS-SAPT0	-25.86	-17.40	-14.10	-13.23

Table S13: PSI4 supermolecular counterpoise-corrected interaction energies for the dimer structures optimized within the B3LYP-GD3/cc-pVDZ approach, [kcal/mol]. Pochyłe energie uzyskane w plikach o nazwie 2.k.hb - w zasadzie ta sama struktura

Dimer	lS	sS	lhb	lhb-B3LYP
		aug-cc-pVTZ #bf 3128		
B3LYP	4.48	16.85	-3.53	-7.29
B3LYP-D2	-27.15	-17.77	-15.29	-13.48
B3LYP-D3	-24.67	-15.78	-15.39	-13.98
B3LYP-D3BJ	-25.99	-17.99	-14.84	-13.32
B3LYP-CHG	-18.59	-10.23	-12.50	-12.04
B97-D2	-26.18	-17.62	-14.09	-12.05
B97-D3	-23.82	-14.75	-14.32	-12.63
B97-D3BJ	-26.49	-18.92	-14.36	-12.29
B2PLYP	-11.07	-0.53	-8.97	-10.29
B2PLYP-D2	-27.64	-18.67	-15.13	-13.53
B2PLYP-D3	-26.22	-17.27	-15.30	-13.90
B2PLYP-D3BJ	-26.75	-18.66	-14.87	-13.46
DSD-BLYP	-29.39	-20.80	-15.55	-13.79
DSD-PBEPBE	-27.92	-18.97	-14.77	-13.11
DSD-PBEP86	-27.93	-19.15	-15.09	-13.50
wB97X-D	-27.08	-19.64	-14.59	-12.40
DLDF	19.20	29.01	3.19	-3.24
DLDF+D	-22.86	-16.04	-11.94	-11.18
DLDF+D09	-24.12	-19.02	-11.33	-10.91
M05	-6.37	3.58	-7.98	-9.95
M05-2X	-18.82	-7.56	-11.40	-11.51
M05-2X-D3	-24.76	-14.99	-14.82	-13.77
M05-D3	-19.92	-12.07	-14.20	-13.68
MP2	-35.88	-28.90	-16.88	-14.25
MP2C	-25.15	-16.36	-15.01	-13.90

Table S14: PSI4 supermolecular uncorrected interaction energies for the dimer structures optimized within the B3LYP-GD3/cc-pVDZ approach, [kcal/mol]

Dimer	lS	sS	lhb	lhb-B3LYP
		cc-pVDZ #bf 872		
B3LYP	-2.90	10.79	-8.26	-11.98
B3LYP-D2	-34.54	-23.83	-20.03	-18.17
B3LYP-D3	-32.06	-21.84	-20.13	-18.68
B3LYP-D3BJ	-33.38	-24.05		-18.01
B3LYP-CHG	-25.98	-16.29	-17.23	-16.73
B97-D2	-33.34	-23.72	-18.46	-16.48
B97-D3	-30.98	-20.85	-18.70	-17.06
B97-D3BJ	-33.65	-25.02	-18.74	-16.72
B2PLYP	-16.97	-4.78	-12.96	-14.41
B2PLYP-D2	-33.54	-22.92	-19.12	-17.66
B2PLYP-D3	-32.12	-21.53	-19.29	-18.03
B2PLYP-D3BJ	-32.65	-22.92	-18.85	-17.59
DSD-BLYP	-34.62	-24.28	-19.11	-17.52
DSD-PBEPBE	-34.27	-23.16	-18.47	-16.80
DSD-PBEP86	-33.57	-23.86	-18.61	-17.09
wB97X-D	-33.71	-25.21	-18.38	-16.04
DLDF	12.46	23.84	-0.95	-6.72
DLDF+D	-29.59	-21.22	-16.08	-14.66
DLDF+D09	-30.84	-24.19	-15.47	-14.39
M05	-14.62	-3.05	-12.69	-14.35
M05-2X	-25.33	-13.08	-15.06	-15.22
M05-2X-D3	-31.28	-20.51	-18.49	-17.47
M05-D3	-28.17	-18.72	-18.91	-18.07
MP2	-38.84	-29.81	-19.40	-17.28

Table S15: PSI4 supermolecular uncorrected interaction energies for the dimer structures optimized within the B3LYP-GD3/cc-pVDZ approach, [kcal/mol]

Dimer	lS	sS	lhb	lhb-B3LYP
		cc-pVTZ #bf 872		
B3LYP	1.67	14.21	-5.13	-8.81
B3LYP-D2	-29.96	-20.41	-16.90	-15.00
B3LYP-D3	-27.48	-18.42	-17.00	-15.50
B3LYP-D3BJ	-28.80	-20.63	-16.45	-14.84
B3LYP-CHG	-21.40	-12.86	-14.10	-13.55
B97-D2	-28.83	-20.11	-15.63	-13.59
B97-D3	-26.47	-17.24	-15.86	-14.18
B97-D3BJ	-29.14	-21.41	-15.91	-13.84
B2PLYP	-15.82	-5.12	-11.17	-12.13
B2PLYP-D2	-32.39	-23.26	-17.33	-15.37
B2PLYP-D3	-30.97	-21.86	-17.50	-15.74
B2PLYP-D3BJ	-31.50	-23.25	-17.06	-15.30
DSD-BLYP	-35.34	-26.61	-18.10	-15.79
DSD-PBEPBE	-34.12	-25.04	-17.37	-15.16
DSD-PBEP86	-34.06	-25.14	-17.66	-15.51
wB97X-D	-29.56	-21.90	-15.99	-13.69
DLDF	16.02	25.95	1.64	-4.51
DLDF+D	-26.03	-19.11	-13.49	-12.44
DLDF+D09	-27.29	-22.08	-12.87	-12.18
M05	-9.19	1.12	-9.73	-11.54
M05-2X	-21.43	-10.08	-12.69	-12.70
M05-2X-D3	-27.37	-17.50	-16.11	-14.95
M05-D3	-22.74	-14.54	-15.95	-15.27
MP2	-40.72	-33.61	-19.08	-15.84

Table S16: PSI4 supermolecular uncorrected interaction energies for the dimer structures optimized within the B3LYP-GD3/cc-pVDZ approach, [kcal/mol]

Dimer	lS	sS	lhb	lhb-B3LYP
		jun-cc-pVDZ #bf 872		
B3LYP	1.51	13.67	-4.58	-8.26
B3LYP-D2	-30.13	-20.95	-16.35	-14.45
B3LYP-D3	-27.65	-18.96	-16.45	-14.95
B3LYP-D3BJ	-28.96	-21.18	-15.90	-14.29
B3LYP-CHG	-21.57	-13.41	-13.55	-13.00
B97-D2	-29.47	-21.15	-15.21	-12.97
B97-D3	-27.11	-18.28	-15.44	-13.56
B97-D3BJ	-29.78	-22.45	-15.49	-13.22
B2PLYP	-16.76	-7.25	-11.31	-11.79
B2PLYP-D2	-33.34	-25.40	-17.47	-15.03
B2PLYP-D3	-31.91	-24.00	-17.64	-15.40
B2PLYP-D3BJ	-32.45	-25.39	-17.21	-14.96
DSD-BLYP	-36.85	-29.81	-18.70	-15.65
DSD-PBEPBE	-36.40	-29.09	-18.21	-15.13
DSD-PBEP86	-35.87	-28.69	-18.38	-15.45
wB97X-D	-30.72	-23.67	-15.82	-13.43
DLDF	16.43	26.21	2.07	-4.11
DLDF+D	-25.62	-18.85	-13.06	-12.04
DLDF+D09	-26.87	-21.82	-12.45	-11.77
M05	-10.93		-9.63	-11.28
M05-2X	-22.96	-12.35	-12.65	-12.59
M05-2X-D3	-28.90	-19.78	-16.07	-14.85
M05-D3	-24.48		-15.85	-15.01
MP2	-46.52	-42.25	-21.56	-16.73

Table S17: PSI4 supermolecular uncorrected interaction energies for the dimer structures optimized within the B3LYP-GD3/cc-pVDZ approach, [kcal/mol]

Dimer	lS	sS	lhb	lhb-B3LYP
		jun-cc-pVTZ #bf 872		
B3LYP	3.60	15.84	-3.87	-7.54
B3LYP-D2	-28.04	-18.79	-15.64	-13.73
B3LYP-D3	-25.56	-16.80	-15.74	-14.24
B3LYP-D3BJ	-26.87	-19.01	-15.19	-13.57
B3LYP-CHG	-19.48	-11.24	-12.84	-12.29
B97-D2	-27.15	-18.74	-14.45	-12.34
B97-D3	-24.80	-15.87	-14.68	-12.93
B97-D3BJ	-27.47	-20.03	-14.73	-12.59
B2PLYP	-16.58	-6.88	-11.04	-11.63
B2PLYP-D2	-33.15	-25.02	-17.20	-14.87
B2PLYP-D3	-31.73	-23.62	-17.37	-15.25
B2PLYP-D3BJ	-32.26	-25.01	-16.94	-14.80
DSD-BLYP	-37.92	-30.62	-18.76	-15.86
DSD-PBEPBE	-37.03	-29.42	-18.21	-15.36
DSD-PBEP86	-36.91	-29.47	-18.48	-15.71
wB97X-D	-28.20	-20.93	-15.14	-12.81
DLDF	17.66	27.22	2.68	-3.59
DLDF+D	-24.38	-17.84	-12.45	-11.52
DLDF+D09	-25.64	-20.81	-11.84	-11.25
M05	-7.46	2.33	-8.54	-10.39
M05-2X	-20.11	-9.06	-11.90	-11.91
M05-2X-D3	-26.05	-16.48	-15.32	-14.16
M05-D3	-21.01	-13.33	-14.76	-14.11
MP2	-43.63	-38.05	-19.84	-15.94

Table S18: PSI4 supermolecular uncorrected interaction energies for the dimer structures optimized within the B3LYP-GD3/cc-pVDZ approach, [kcal/mol]

Dimer	lS	sS	lhb	lhb-B3LYP
		aug-cc-pVDZ #bf 872		
B3LYP	0.06	11.73	-5.62	-8.73
B3LYP-D2	-31.58	-22.89	-17.39	-14.93
B3LYP-D3	-29.10	-20.90	-17.49	-15.43
B3LYP-D3BJ	-30.41	-23.11	-16.94	-14.76
B3LYP-CHG	-23.02	-15.34	-14.59	
B97-D2	-30.87	-23.00	-16.18	-13.39
B97-D3	-28.52	-20.13	-16.41	-13.98
B97-D3BJ	-31.19	-24.29	-16.46	-13.64
B2PLYP	-20.95	-11.76	-13.33	-13.03
B2PLYP-D2	-37.53	-29.90	-19.50	-16.27
B2PLYP-D3	-36.10	-28.50	-19.66	-16.64
B2PLYP-D3BJ	-36.64	-29.89	-19.23	-16.20
DSD-BLYP	-42.78	-35.96	-21.37	-17.35
DSD-PBEPBE	-42.02	-34.88	-20.77	-16.68
DSD-PBEP86	-41.61	-34.62	-20.99	-17.07
wB97X-D	-32.44	-25.85	-17.05	-13.86
DLDF	14.46	23.69	0.78	-4.80
DLDF+D	-27.59	-21.37	-14.35	-12.73
DLDF+D09	-28.84	-24.34	-13.74	-12.47
M05	-12.02	-2.83	-10.47	-11.50
M05-2X	-25.42	-15.33	-14.18	-13.18
M05-2X-D3	-31.37	-22.76	-17.61	-15.43
M05-D3	-25.57	-18.50	-16.69	-15.22
MP2	-55.48	-51.16	-25.39	-19.34

Table S19: PSI4 supermolecular uncorrected interaction energies for the dimer structures optimized within the B3LYP-GD3/cc-pVDZ approach, [kcal/mol]

Dimer	lS	sS	lhb	lhb-B3LYP
		aug-cc-pVTZ #bf 872		
B3LYP	3.39	15.68	-4.05	-7.72
B3LYP-D2	-28.24	-18.94	-15.82	-13.91
B3LYP-D3	-25.76	-16.95	-15.92	-14.42
B3LYP-D3BJ	-27.07	-19.16	-15.37	-13.75
B3LYP-CHG	-19.68	-11.40	-13.02	-12.47
B97-D2	-27.32	-18.84	-14.62	-12.50
B97-D3	-24.97	-15.97	-14.85	-13.09
B97-D3BJ	-27.64	-20.14	-14.90	-12.74
B2PLYP	-17.18	-7.44	-11.86	-12.38
B2PLYP-D2	-33.76	-25.58	-18.02	-15.63
B2PLYP-D3	-32.34	-24.19	-18.19	-16.00
B2PLYP-D3BJ	-32.87	-25.57	-17.76	-15.56
DSD-BLYP	-38.84	-31.52	-20.02	-17.01
DSD-PBEPBE	-37.97	-30.37	-19.55	-16.56
DSD-PBEP86	-37.85	-30.42	-19.80	-16.90
wB97X-D	-28.41	-21.07	-15.31	-12.93
DLDF	17.26	26.84	2.32	-3.90
DLDF+D	-24.79	-18.22	-12.80	-11.84
DLDF+D09	-26.04	-21.19	-12.19	-11.57
M05	-8.18	1.61	-8.93	-10.65
M05-2X	-20.10	-8.99	-12.06	-12.12
M05-2X-D3	-26.04	-16.42	-15.48	-14.37
M05-D3	-21.74	-14.05	-15.15	-14.38
MP2	-44.71	-38.77	-20.92	-16.83

Table S20: PSI4 basis set superposition error for the dimer structures optimized within the B3LYP-GD3/cc-pVDZ approach, [kcal/mol]

Dimer	lS	sS	lhb	lhb-B3LYP
		cc-pVDZ #bf 872		
B3LYP	7.83	6.41	5.12	5.06
B3LYP-D2	7.83	6.41	5.12	5.06
B3LYP-D3	7.83	6.41	5.12	5.06
B3LYP-D3BJ	7.83	6.41	5.12	5.06
B3LYP-CHG	7.83	6.41	5.12	5.06
B97-D2	7.29	6.11	4.70	4.76
B97-D3	7.29	6.11	4.70	4.76
B97-D3BJ	7.29	6.11	4.70	4.76
B2PLYP	10.44	8.91	6.11	5.47
B2PLYP-D2	10.44	8.91	6.11	5.47
B2PLYP-D3	10.44	8.91	6.11	5.47
B2PLYP-D3BJ	10.44	8.91	6.11	5.47
DSD-BLYP	11.81	10.22	6.55	5.57
DSD-PBEPBE	11.06	9.55	6.04	5.15
DSD-PBEP86	11.37	9.84	6.23	5.28
wB97X-D	6.92	5.53	4.29	4.01
DLDF	7.46	6.00	4.53	4.00
DLDF+D	7.46	6.00	4.53	4.00
DLDF+D09	7.46	6.00	4.53	4.00
M05	7.64	6.16	4.79	4.55
M05-2X	6.88	5.48	4.30	4.26
M05-2X-D3	6.88	5.48	4.30	4.26
M05-D3	7.64	6.16	4.79	4.55
MP2	15.53	13.81	8.08	6.36

Table S21: PSI4 basis set superposition error for the dimer structures optimized within the B3LYP-GD3/cc-pVDZ approach, [kcal/mol]

Dimer	lS	sS	lhb	lhb-B3LYP
		cc-pVTZ #bf 2000		
B3LYP	2.83	2.69	1.62	1.60
B3LYP-D2	2.83	2.69	1.62	1.60
B3LYP-D3	2.83	2.69	1.62	1.60
B3LYP-D3BJ	2.83	2.69	1.62	1.60
B3LYP-CHG	2.83	2.69	1.62	1.60
B97-D2	2.72	2.59	1.59	1.63
B97-D3	2.72	2.59	1.59	1.63
B97-D3BJ	2.72	2.59	1.59	1.63
B2PLYP	5.89	5.79	2.76	2.30
B2PLYP-D2	5.89	5.79	2.76	2.30
B2PLYP-D3	5.89	5.79	2.76	2.30
B2PLYP-D3BJ	5.89	5.79	2.76	2.30
DSD-BLYP	7.69	7.61	3.37	2.65
DSD-PBEPBE	7.63	7.56	3.28	2.58
DSD-PBEP86	7.71	7.66	3.32	2.60
wB97X-D	2.57	2.47	1.43	1.32
DLDF	2.98	2.82	1.52	1.38
DLDF+D	2.98	2.82	1.52	1.38
DLDF+D09	2.98	2.82	1.52	1.38
M05	2.94	2.80	1.81	1.66
M05-2X	2.67	2.67	1.32	1.25
M05-2X-D3	2.67	2.67	1.32	1.25
M05-D3	2.94	2.80	1.81	1.66
MP2	8.31	8.27	1.79	2.77

Table S22: PSI4 basis set superposition error for the dimer structures optimized within the B3LYP-GD3/cc-pVDZ approach, [kcal/mol]

Dimer	lS	sS	lhb	lhb-B3LYP
	jun-cc-pVDZ #bf 1064			
B3LYP	3.22	3.65	1.36	1.02
B3LYP-D2	3.22	3.65	1.36	1.02
B3LYP-D3	3.22	3.65	1.36	1.02
B3LYP-D3BJ	3.22	3.65	1.36	1.02
B3LYP-CHG	3.22	3.65	1.36	1.02
B97-D2	3.28	3.75	1.34	0.97
B97-D3	3.28	3.75	1.34	0.97
B97-D3BJ	3.28	3.75	1.34	0.97
B2PLYP	8.60	9.77	3.85	2.26
B2PLYP-D2	8.60	9.77	3.85	2.26
B2PLYP-D3	8.60	9.77	3.85	2.26
B2PLYP-D3BJ	8.60	9.77	3.85	2.26
DSD-BLYP	11.75	13.37	5.28	2.99
DSD-PBEPBE	11.49	13.12	5.18	2.93
DSD-PBEP86	11.58	13.20	5.10	2.88
wB97X-D	3.32	3.77	1.42	0.97
DLDF	3.34	3.74	1.35	0.98
DLDF+D	3.34	3.74	1.35	0.98
DLDF+D09	3.34	3.74	1.35	0.98
M05	3.84		1.50	0.97
M05-2X	3.69	4.18	1.51	1.10
M05-2X-D3	3.69	4.18	1.51	1.10
M05-D3	3.84		1.50	0.97
MP2	19.05	21.69	8.71	4.75

Table S23: PSI4 basis set superposition error for the dimer structures optimized within the B3LYP-GD3/cc-pVDZ approach, [kcal/mol]

Dimer	lS	sS	lhb	lhb-B3LYP
		jun-cc-pVTZ #bf 2432		
B3LYP	0.94	1.06	0.38	0.28
B3LYP-D2	0.94	1.06	0.38	0.28
B3LYP-D3	0.94	1.06	0.38	0.28
B3LYP-D3BJ	0.94	1.06	0.38	0.28
B3LYP-CHG	0.94	1.06	0.38	0.28
B97-D2	1.03	1.14	0.40	0.31
B97-D3	1.03	1.14	0.40	0.31
B97-D3BJ	1.03	1.14	0.40	0.31
B2PLYP	5.82	6.63	2.26	1.47
B2PLYP-D2	5.82	6.63	2.26	1.47
B2PLYP-D3	5.82	6.63	2.26	1.47
B2PLYP-D3BJ	5.82	6.63	2.26	1.47
DSD-BLYP	9.00	10.25	3.48	2.25
DSD-PBEPBE	9.50	10.80	3.67	2.40
DSD-PBEP86	9.40	10.70	3.63	2.37
wB97X-D	1.14	1.29	0.55	0.36
DLDF	1.46	1.69	0.51	0.38
DLDF+D	1.46	1.69	0.51	0.38
DLDF+D09	1.46	1.69	0.51	0.38
M05	1.19	1.35	0.57	0.38
M05-2X	1.32	1.53	0.50	0.38
M05-2X-D3	1.32	1.53	0.50	0.38
M05-D3	1.19	1.35	0.57	0.38
MP2	8.72	10.03	3.50	2.04

Table S24: PSI4 basis set superposition error for the dimer structures optimized within the B3LYP-GD3/cc-pVDZ approach, [kcal/mol]

Dimer	lS	sS	lhb	lhb-B3LYP
		aug-cc-pVDZ #bf 1464		
B3LYP	4.68	5.55	2.16	1.46
B3LYP-D2	4.68	5.55	2.16	1.46
B3LYP-D3	4.68	5.55	2.16	1.46
B3LYP-D3BJ	4.68	5.55	2.16	1.46
B3LYP-CHG	4.68	5.55	2.16	1.46
B97-D2	4.67	5.50	2.05	1.34
B97-D3	4.67	5.50	2.05	1.34
B97-D3BJ	4.67	5.50	2.05	1.34
B2PLYP	10.68	12.08	4.69	2.91
B2PLYP-D2	10.68	12.08	4.69	2.91
B2PLYP-D3	10.68	12.08	4.69	2.91
B2PLYP-D3BJ	10.68	12.08	4.69	2.91
DSD-BLYP	14.49	16.22	6.29	3.83
DSD-PBEPBE	14.66	16.38	6.31	3.78
DSD-PBEP86	14.51	16.21	6.28	3.80
wB97X-D	4.72	5.52	2.18	1.34
DLDF	5.25	6.13	2.46	1.62
DLDF+D	5.25	6.13	2.46	1.62
DLDF+D09	5.25	6.13	2.46	1.62
M05	5.05	5.88	2.15	1.23
M05-2X	5.73	6.74	2.52	1.60
M05-2X-D3	5.73	6.74	2.52	1.60
M05-D3	5.05	5.88	2.15	1.23
MP2	21.72	24.16	9.53	5.69

Table S25: PSI4 basis set superposition error for the dimer structures optimized within the B3LYP-GD3/cc-pVDZ approach, [kcal/mol]

Dimer	lS	sS	lhb	lhb-B3LYP
		aug-cc-pVTZ #bf 3128		
B3LYP	1.08	1.16	0.52	0.43
B3LYP-D2	1.08	1.16	0.52	0.43
B3LYP-D3	1.08	1.16	0.52	0.43
B3LYP-D3BJ	1.08	1.16	0.52	0.43
B3LYP-CHG	1.08	1.16	0.52	0.43
B97-D2	1.14	1.22	0.53	0.45
B97-D3	1.14	1.22	0.53	0.45
B97-D3BJ	1.14	1.22	0.53	0.45
B2PLYP	6.11	6.91	2.89	2.09
B2PLYP-D2	6.11	6.91	2.89	2.09
B2PLYP-D3	6.11	6.91	2.89	2.09
B2PLYP-D3BJ	6.11	6.91	2.89	2.09
DSD-BLYP	9.44	10.72	4.46	3.21
DSD-PBEPBE	10.04	11.40	4.78	3.45
DSD-PBEP86	9.91	11.26	4.70	3.39
wB97X-D	1.33	1.43	0.72	0.52
DLDF	1.92	2.17	0.86	0.65
DLDF+D	1.92	2.17	0.86	0.65
DLDF+D09	1.92	2.17	0.86	0.65
M05	1.81	1.97	0.95	0.70
M05-2X	1.28	1.42	0.66	0.60
M05-2X-D3	1.28	1.42	0.66	0.60
M05-D3	1.81	1.97	0.95	0.70
MP2	8.83	9.86	4.03	2.58

Table S26: DFT-SAPT/aug-cc-pVDZ interaction energy components for the dimer structures optimized within the B3LYP-GD3/cc-pVDZ approach, [kcal/mol]

	DFT-SAPT/aug-cc-pVDZ			
Component	lS	sS	lhb	lhb-B3LYP
Electrostatics	-20.38	-6.69	-14.57	-12.07
$E^{(1)}_{pol}$	-20.38	-6.69	-14.57	-12.07
Exchange				
$E^{(1)}_{exch}$	40.12	37.58	22.81	14.40
$E^{(1)}_{exch}(S^2)$	40.12	37.56	22.74	14.35
Induction				
$E^{(2)}_{ind}$	-18.07	-16.8	-10.08	-6.61
$E^{(2)}_{ind-exch}$	15.14	14.3	6.86	3.14
Dispersion				
$E^{(2)}_{disp}$	-46.55	-46.3	-20.36	-20.65
$E^{(2)}_{disp-exch}$	6.46	6.3	2.85	1.97
$E^{(1)}$(tot)	19.74	30.89	8.24	2.33
$E^{(2)}$(tot)	-43.02	-42.54	-20.73	-22.16
$E^{(1)}$(tot)+$E^{(2)}$(tot)	-23.28	-11.65	-12.49	-19.83
$E^{(1)}$(tot)+$E^{(2)}$(tot)+δ(HF)	-26.81	-14.39	-15.19	-21.66

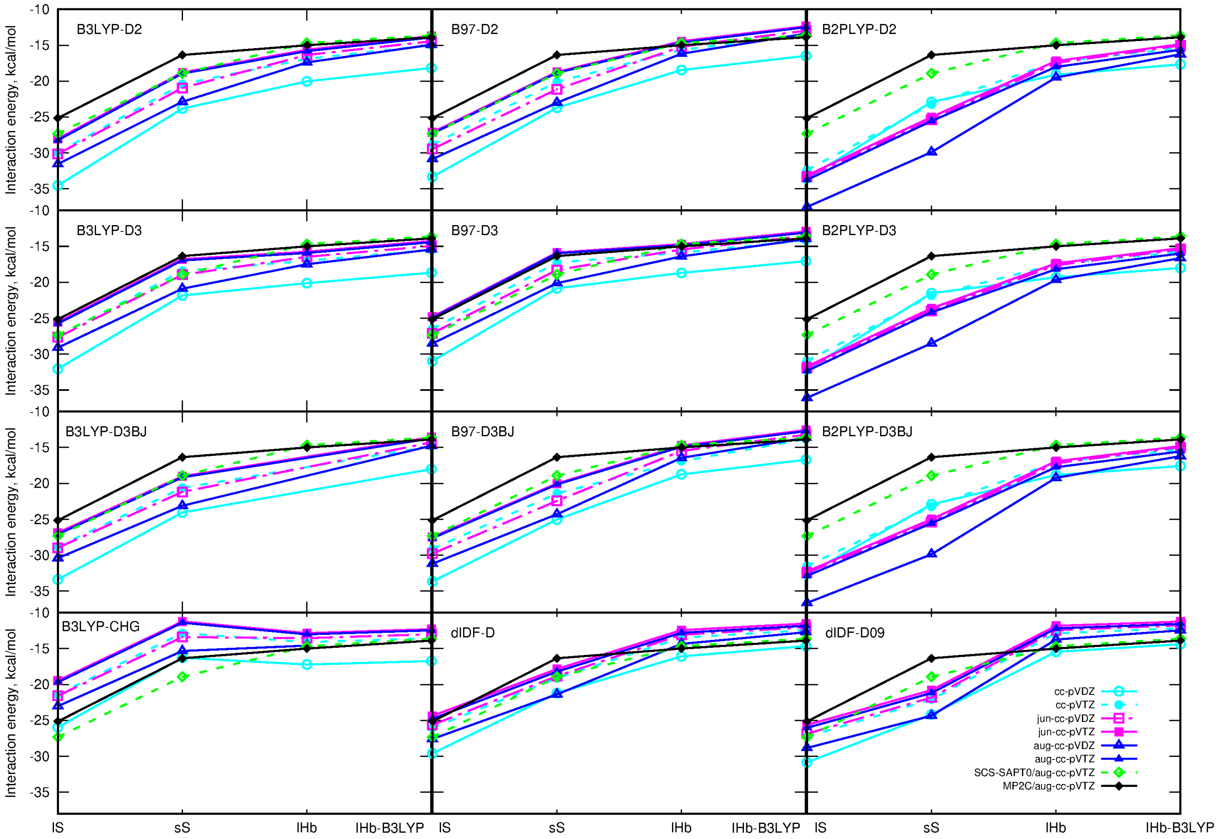

Figure S2: Supermolecular uncorrected interaction energy with various dispersion corrections with respect to the SCS-SAPT0/aug-cc-pVTZ and MP2C/aug-cc-pVTZ data

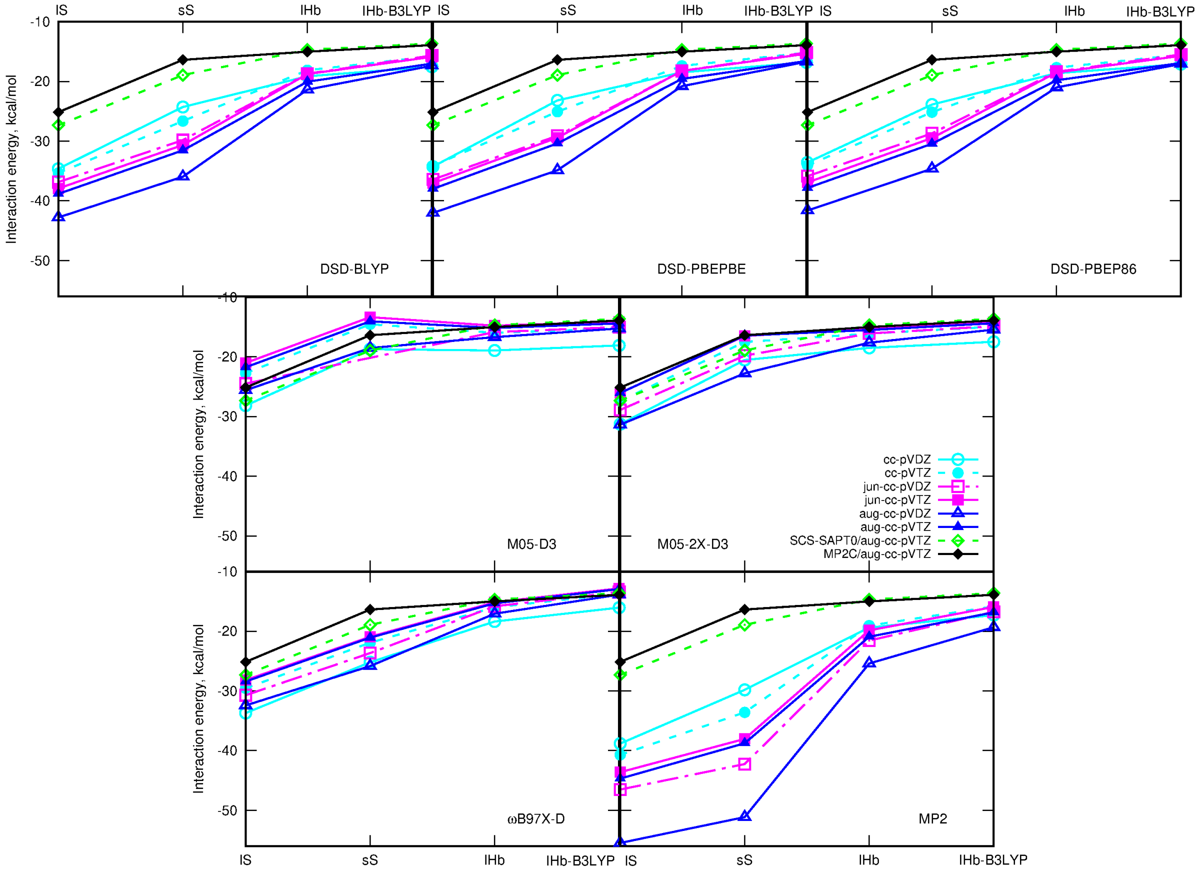

Figure S3: Supermolecular uncorrected interaction energy with various DSD functionals with respect to the SCS-SAPT0/aug-cc-pVTZ and MP2C/aug-cc-pVTZ data

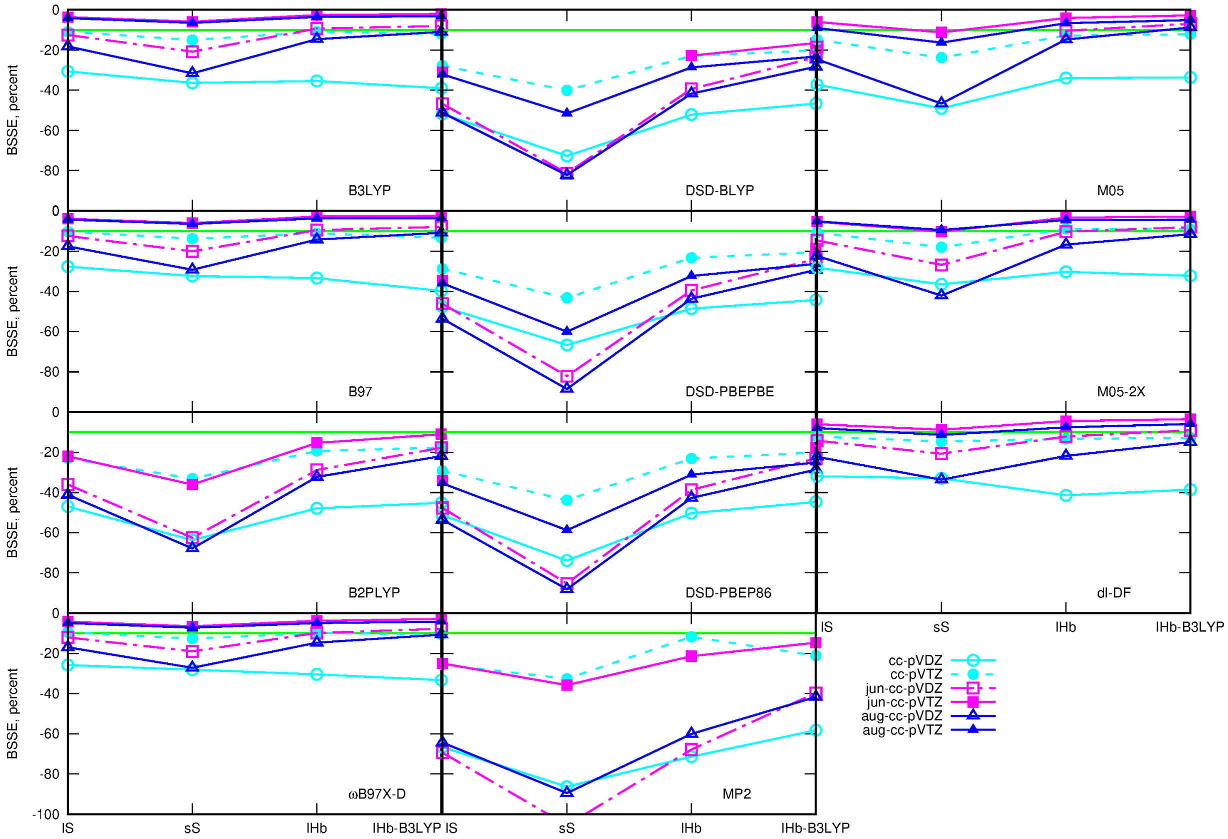

Figure S4: Percentage of the basis set superposition error with various functionals and basis sets with respect to the total supermolecular interaction energy obtained with the same approximation. Horizontal solid green line depicts the level of 10%.
